# Comparing Force Field Treatments in QM/MM Studies of the SARS-CoV‑2 RNA-Dependent RNA Polymerase (RdRp) Mechanism

**DOI:** 10.1021/acs.jctc.5c01399

**Published:** 2025-11-24

**Authors:** Maite Roca, Yazdan Maghsoud, G. Andrés Cisneros, Katarzyna Świderek, Vicent Moliner

**Affiliations:** † BioComp Group. Institute of Advanced Materials (INAM), 16748 Universitat Jaume I, Castelló de la Plana 12071, Spain; ‡ Department of Chemistry and Biochemistry, 12335 The University of Texas at Dallas, Richardson, Texas 75080, United States; § Department of Biochemistry and Molecular Pharmacology, Baylor College of Medicine, Houston, Texas 77030, United States; ∥ Department of Physics, The University of Texas at Dallas, Richardson, Texas 75080, United States

## Abstract

Molecular simulations have been instrumental in elucidating the SARS-CoV-2 lifecycle, thereby supporting the design and development of antiviral therapies and diagnostic tools for COVID-19. Here, the molecular mechanism of the SARS-CoV-2 RNA-dependent RNA polymerase (RdRp), a potential target for antiviral drugs to treat COVID-19, was explored based on QM/MM simulations with fixed-charge and polarizable force fields (cFF and pFF, respectively). The free energy perturbation (FEP) method allowed exploring the free energy landscape of the enzymatic reaction mechanism, addressing key questions about the initial deprotonation of the 3′-OH group of the terminal nucleotide before a nucleophilic attack on the incoming nucleotide takes place. Indeed, among the five mechanisms explored, the most favorable was identified as a three-step process. The first step consists of a proton transfer from the 3′-OH group of the terminal nucleotide to a hydroxide group coordinated with an Mg^2+^ ion. Subsequently, the O3′ atom nucleophilically attacks the Pα atom of the incoming ATP. Finally, a proton is transferred from the water molecule formed in the first step to the γ-phosphate group of the pyrophosphate leaving group, regenerating the Mg^2+^-coordinated hydroxide group. This mechanism was found to be exergonic, with the rate-determining step being the nucleophilic attack, having a free energy barrier of 15.2 kcal mol^–1^. Both cFF and pFF yield consistent energetic and geometrical descriptions of the full RdRp-catalyzed reaction. Noncovalent interaction (NCI) and electron localization function (ELF) analyses provide insights into the electronic evolution during the reaction, showing strong polarization on electronic basins associated with the reactive oxygens O3′ and O3α. Together, findings contribute to a deeper understanding of the RdRp mechanism, which could aid in the discovery of new antiviral inhibitors.

## Graphical abstract

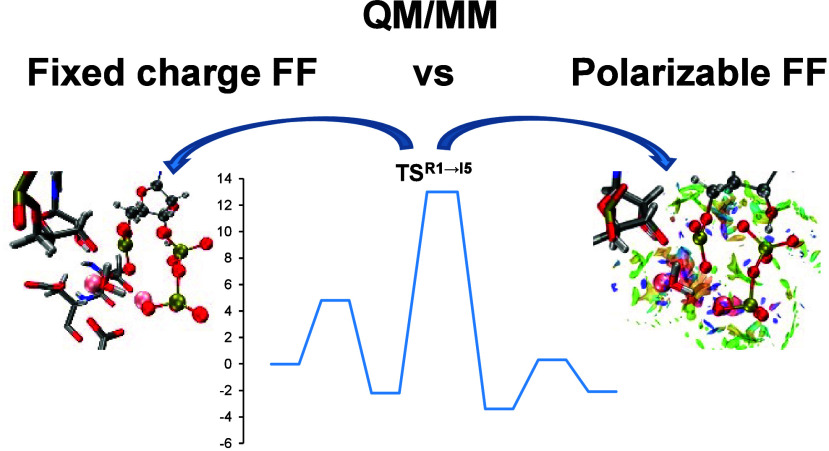

## 1 Introduction

In December 2019, a novel severe acute respiratory syndrome coronavirus-2 (SARS-CoV-2) emerged in China and rapidly spread worldwide, leading to the COVID-19 pandemic that caused over 7 million deaths.^1^ This virus spreads mainly through respiratory droplets and can cause symptoms ranging from asymptomatic to severe respiratory failure. Like other coronaviruses, SARS-CoV-2 primarily infects the respiratory and gastrointestinal tracts, producing symptoms such as fever, cough, myalgia, headache, and even diarrhea.^2,3^ In severe cases, it can lead to respiratory failure, septic shock, and/or multiple organ dysfunction or failure resulting in death.^4^ Global responses, including lockdowns and social distancing, profoundly affected economic and social life. Although significant progress has been made in understanding and controlling the virus, its rapid mutation continues to pose challenges, requiring ongoing research. In this context, molecular simulations of enzyme reactivity have proven valuable for elucidating the SARS-CoV-2 lifecycle.^5,6^ In particular, focusing on two key proteins: the main protease (Mpro/3CLpro), a target for antiviral drugs, and the spike protein, a target for vaccine design.^6^

The virus enters the host cells through direct interaction between the viral spike protein and the cellular receptor angiotensin-converting enzyme 2 (ACE2). Inside the cell, viral genome replication and the transcription of its genes are mediated by the viral replication complex, which includes the RNA-dependent RNA polymerase (RdRp). Therefore, RdRp represents another potential target for blocking the virus, in addition to Mpro and spike proteins, and a proposed antiviral strategy involves using nucleoside or nucleotide analogs to inhibit viral genome replication.^7^ Numerous efforts have focused on designing effective inhibitors of RdRp. However, the mechanisms of nucleoside-triphosphate binding, nucleotide activation and incorporation, and the complete reaction mechanism catalyzed by RdRp are under debate.^8–10^

It is generally accepted that RNA synthesis by polymerases involves the template-guided addition of ribonucleoside triphosphate (rNTP) to the O3′ atom of the terminal nucleotide of the RNA strand via a nucleotidyl transfer reaction (see Scheme 1). Nevertheless, the 3′-OH group of the terminal nucleotide should be previously deprotonated to form the activated nucleophile, which then attacks the Pα atom of the incoming nucleotide in an S_N_2-like reaction to form a new bond between the O3′ and Pα atom, thereby extending the RNA strand by one nucleotide. Simultaneously, the phosphodiester bond between the α- and β-phosphates must be broken, releasing pyrophosphate.^11^ Thus, it remains unclear which residue could act as a base to abstract the proton from the 3′–OH group of the terminal nucleotide, as well as the timing of the different breaking and forming bonds. In this case, molecular simulations can provide information to assist in understanding the reaction mechanism catalyzed by RdRp from SARS-CoV-2, including the lack of consensus regarding the deprotonation mechanism.^8–10^

**Scheme 1:**
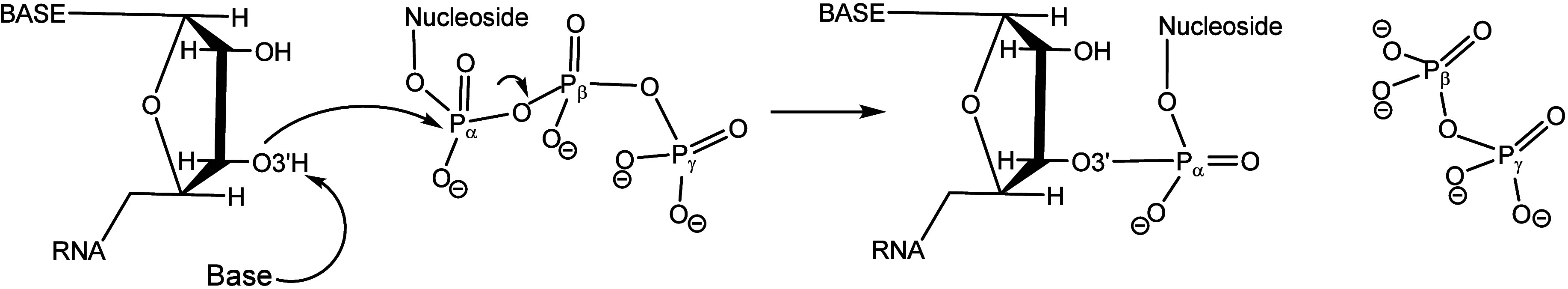
Nucleotide Transfer Reaction for the Inclusion of Nucleoside-Triphosphate on the Nascent RNA Strand, Preceded by Proton Abstraction from a Residue That Functions As a Base

Although the crystal structures of DNA and RNA polymerases differ in overall architecture, their active sites share conserved catalytic motifs and metal-ion coordination geometries. The active sites usually contain two metal ions, each coordinated to an acidic residue (Asp or Glu), as well as to some water molecules and the triphosphate group of the incoming nucleotide. Several computational studies of the catalytic mechanisms of DNA polymerase have been reported in the literature.^12–14^ Using computational approaches, it is possible to elucidate key aspects of the chemical reaction that have eluded experiments, particularly the identity of the base that deprotonates the O3′ atom of the 3′-OH group of the terminal nucleotide. This O3′ atom is coordinated to a metal ion; thus, it is believed that it lowers its p*K*a. For the DNA Pol β system studied by Wilson et al., it was proposed that a conserved aspartate residue bound to a metal ion served as the general base for the deprotonation step, based on ONIOM quantum mechanics/molecular mechanics (QM/MM) calculations.^15^ Warshel and co-workers used free energy perturbation/empirical valence bond (FEP/EVB) calculations to investigate alternative pathways for T7 DNA Pol, including proton transfer to bulk solvent and to one of the nonbridging oxygen atoms of the Pα of the incoming nucleotide. However, proton transfer to the catalytic residue (Asp654) was the most energetically favorable pathway.^16^ Comparably, other systems that contained acidic residues close to the O3′ atom were shown to be the most plausible base by QM/MM studies.^17–19^ In another study of DNA Pol *I* using molecular dynamics (MD) simulations, His829 was proposed as a potential proton acceptor.^20^

Zhang and co-workers proposed an alternative mechanism, a water-mediated, substrate-assisted mechanism, based on studies of other DNA polymerases.^21–23^ Maghsoud et al.^14^ investigated the characteristics of a three-metal-ion active site, the electrostatic effects of the third metal ion, and the influence of the incoming nucleotide’s charge accumulation on the catalytic reaction of the DNA Pol κ. It was initially proposed that the α-phosphate of the incoming nucleotide acts as the general base to abstract the proton; however, the proton is not directly transferred to it. Instead, the reaction occurs through a water molecule.^21^ Subsequently, the proton on the α-phosphate is reoriented toward the γ-phosphate and is transferred via a second water molecule. Then, the nucleophilic in-line attack of the O3′ atom of the primer 3′-terminal on the α-phosphate occurs, accompanied by a synchronous proton transfer from the γ-phosphate to the β-phosphate via a water molecule.^21^ This mechanism accounts for not only the deprotonation of the O3′ atom but also the protonation of the pyrophosphate leaving group, which is considered to be required for its release.^24^ A variation of this mechanism was proposed in other studies of DNA polymerases, wherein the base to abstract the proton from the 3′-OH group of the terminal nucleotide is γ-phosphate and not α-phosphate.^22,23^ QM/MM calculations from Salahub and co-workers demonstrated that the 3′-H of RNA Pol II is transferred to one oxygen atom of the α-phosphate of the incoming nucleotide either directly or indirectly (through a water molecule or an Asp residue present in the active side), upon which the O3′-Pα is formed and the Pα–Oαβ bond is weakened. Subsequently, the 3′-H is transferred to the Oαβ atom that bridges the α and β-phosphate, leading to the release of pyrophosphate.^25^ Schlick and co-workers proposed a similar water-mediated mechanism for DNA Pol β, where the proton-transfer steps take place from the 3′-OH group of the terminal nucleotide to pyrophosphate, via active site water and Asp190, in conjunction with a nucleophilic attack at the Pα center.^26^ Alternatively, De Vivo and co-workers proposed a self-activated mechanism for DNA and RNA polymerases that consists of the activation of the 3′-OH nucleophile by the intramolecular hydrogen bond between the 3′-OH and the β-phosphate group of the newly incorporated nucleotide, which facilitates the deprotonation of the 3′-OH group.^27^

Other computational studies of DNA polymerases demonstrated the feasibility of the proton transfer from the 3′–OH group of the terminal nucleotide to a deprotonated water molecule coordinated with a metal ion (OH^–^ coordinated with Mg^2+^)^28–30^ or, in the case of RNA Pol II, to a bulk OH^–^ hydrogen bonded to the terminal RNA and near Mg^2+^.^31–34^ Moreover, several studies propose that multisubunit RNA polymerases contain a histidine in the trigger loop that can serve as a general acid to protonate the pyrophosphate leaving group.^31,33–35^

In studying the reaction mechanism of RdRp from SARS-CoV-2, leading to the incorporation of a nucleotide into the nascent viral RNA strand, Bignon and Monari conducted DFT/MM-MD computations coupled with 2D umbrella sampling.^36^ The simulations were carried out using the Terachem program,^37^ interfaced with Amber16.^38^ The QM calculations were performed at the density functional theory (DFT) level using the ωB97x-D exchange–correlation functional and the double-ζ 6–31G basis set. In this contribution, due to the absence of a base to enforce a proton shuttle from the O3′ atom of the terminal nucleotide of the RNA strand, they kept O3′ deprotonated and focused on the reaction step involving the attack of the O3′ atom of the terminal nucleotide of the RNA strand on the nucleotide to be bound. For the last step of the reaction—the protonation of the pyrophosphate leaving group—they observed that the SARS-CoV-2 polymerase active site differs slightly from those of other RNA polymerases, lacking a nearby histidine. Thus, they proposed that a lysine residue could assist in protonating the pyrophosphate group.^36^

More recently, Orozco and co-workers studied the reaction mechanism of RdRp from SARS-CoV-2.^39^ They conducted QM/MM-MD simulations to determine the minimum free-energy paths (MFEPs) using the string method. The MFEPs were computed at the DFTB3/MM level, and the potential of mean force (PMF) profiles were subsequently corrected at the B3LYP/6–311++G** level. The QM/MM calculations were performed with electrostatic embedding using the AMBER 19 program.^40^ High-level corrections were carried out with Gaussian 16.^41^ Nevertheless, their initial system was constructed using homology modeling before the release of experimental structures for the SARS-CoV-2 RdRp. They proposed a reaction mechanism that consists of two steps. The first step involves proton transfer from the O3′ of the just-incorporated terminal nucleotide to the γ-phosphate of the previously formed pyrophosphate leaving group. The second step is the nucleophilic attack of the O3′ atom of the just incorporated terminal nucleotide to the Pα atom of the incoming nucleotide. Based on their findings, the second step is identified as the rate-limiting step, proceeding through a concerted associative transition state (TS), where the breaking and forming bond lengths are found to be similarly elongated.^39^

Despite these previous studies, significant questions about the mechanism remain unresolved. With this in mind, to unveil the enzymatic reaction mechanism catalyzed by RdRp from SARS-CoV-2, free energy landscapes at a high level of theory must be explored. In addition, for the case under study here, several mechanistic routes are possible, and all should be analyzed. To make this study affordable, we conducted QM/MM calculations to explore potential energy surfaces (PES) and computed free energy profiles at the ωB97X-D/MM level by the use of free energy perturbation method (FEP) to gain more accurate insights into the SARS-CoV-2 RdRp reaction mechanism. In addition, we took the opportunity to study an essential technical question: whether the use of polarizable force fields (pFF), in contrast to conventional fixed-charge force fields (cFF), can have a relevant impact on the results of QM/MM computer simulations. We have investigated how charge distributions and polarization effects calculated by a pFF influence the energy profile and critical points. A more accurate understanding of this mechanism and an analysis of the TS’s structure should facilitate the discovery of new antiviral inhibitors targeting emerging RNA viruses.

## 2 Methods

### 2.1 MM MD Simulations

The detailed procedures for structure preparation and MD simulation are described elsewhere.^42^ Briefly, the SARS-CoV-2 RdRp model was constructed using the cryo-EM structure from Hillen et al.^43^ (PDB 6YYT), with missing segments completed from Gao et al.^44^ (PDB 7BTF). As shown in Figure 1, the structure is composed of the viral nonstructural protein 12 (nsp12, chain A), nsp8 (chains B and D), and nsp7 (chain C), and more than two turns of RNA template. The active-site cleft of nsp12 binds to the first turn of RNA and mediates RdRp activity with conserved residues. The active site was modeled based on Zamyatkin et al.^45^ (PDB 3H5Y). The system was solvated in TIP3P^46^ water with K^+^ counterions for neutralization, and Amber-ff14SB,^47^ RNA-χOL3,^48^ and ZAFF^49^ FF were used for the protein, RNA, and Zn ^2+^ cations, respectively, with additional parameters for Mg^2+^ ions.^50^ Nucleotide parameters were obtained from pyRED^51^ and Meagher et al.^52^ MD simulations were performed in triplicate using AMBER18,^53^ with heating, relaxation, and production runs in the NPT ensemble at 300 K for 350 ns. The Langevin thermostat^54–56^ and Berendsen barostat^57^ were applied, with PME^58^ for long-range electrostatics and a 9Å cutoff for nonbonded interactions.

**Figure 1:**
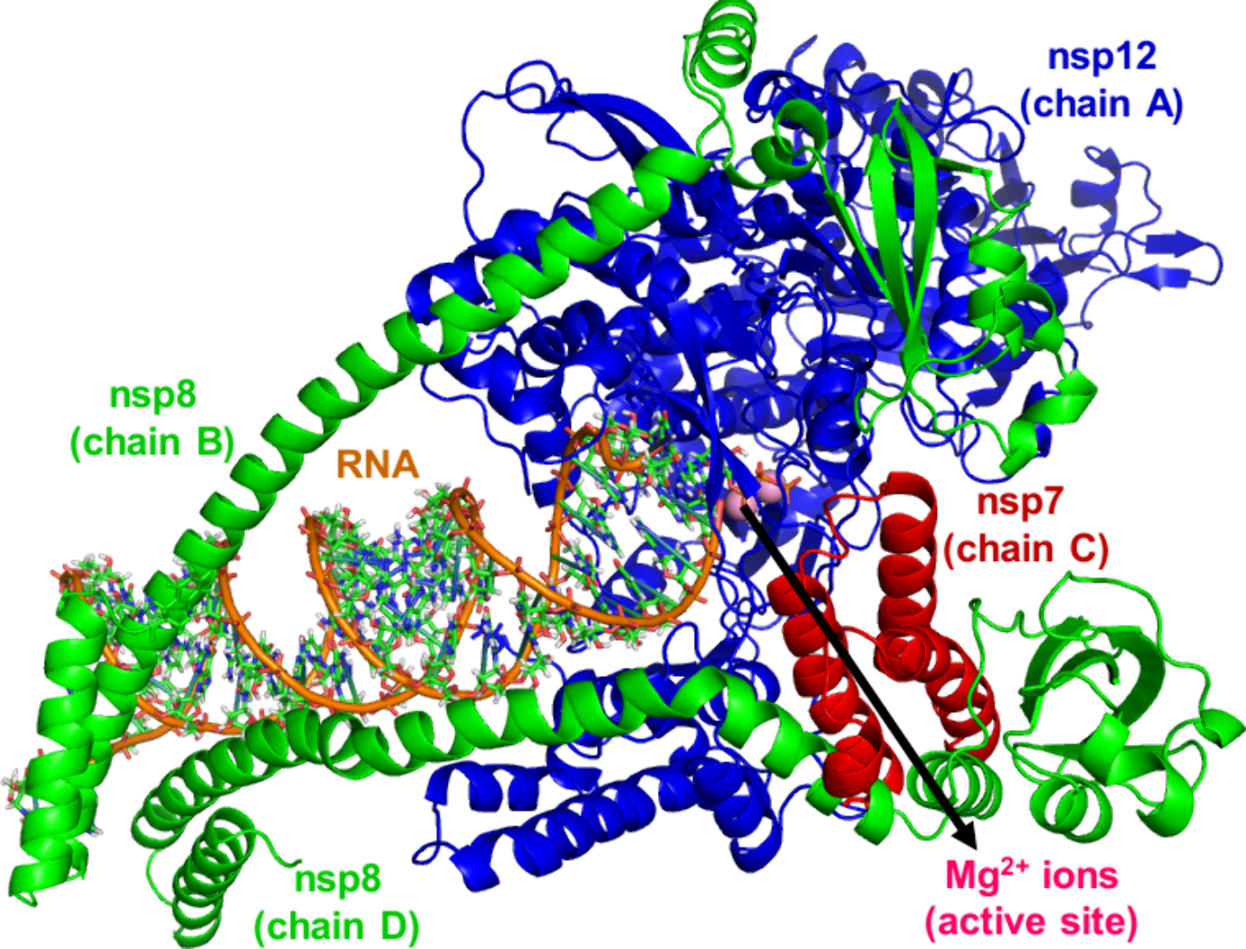
Structure of nsp12 (in blue), nsp8 (in green), and nsp7 (in red) subunits of RdRp with RNA template. The pink spheres depict the two Mg^2+^ ions in the active site.

### 2.2 QM/MM MD Simulations

After relaxing the full system with adenosine triphosphate (ATP) in the active site as the incoming nucleotide, using classical MD simulations by Naseem-Khan et al.,^42^ the system was simplified by removing all water molecules beyond a 40 Å sphere around ATP. The truncated system consists of 1371 amino acid residues, 54 nucleotides, two Zn^2+^ ions, two Mg^2+^ ions, the ATP molecule, 61 K^+^ ions to neutralize the system, and 7402 water molecules (see Figure S1 of the Supporting Information). All residues located further than 20 Å from the ATP molecule were fixed in the optimizations and MD simulations using QM/MM potentials. The structure was partitioned into a QM and an MM region. The QM region, consisting of 93 atoms, contains the ATP, part of the 3′-terminal nucleotide of the RNA, the Mg^2+^ ions, and the side chains of the residues involved in the coordination spheres of the Mg^2+^ ions (see Figure S2 in the Supporting Information). The two Mg^2+^ ions are coordinated by the phosphate groups of the incoming triphosphate nucleotide, ATP in this particular case, as well as Asp618, Tyr619, Asp760, Asp761, a water molecule, and the O3′ atom of the RNA strand. The QM region, was described with the AM1d^59,60^ and the density functional theory (DFT) ωB97X-D^61^ Hamiltonian with def2-SVP^62^ basis set. The AMBER ff14SB^63^ was used to describe the residues of the protein, AMBER force field ff99 ^64^ with the parmbsc0 ^65^ and parmχOL3 ^66^ dihedral modifications, supplemented by van der Waals parameters for phosphates^67^ was used for the RNA, ZAFF^49^ FF for Zn^2+^ ions, and the TIP3P^46^ force field was selected to describe the water molecules and the K^+^ ions. Parameters for Mg^2+^ were adapted from Villa and co-workers.^50^ Hydrogen link atoms were placed in the QM–MM frontier bonds. A switched cutoff from 14.5 to 16 Å was employed for all nonbonded MM interactions.

First, the system was minimized using a conjugate gradient algorithm, followed by short QM/MM MD simulations with AM1d/AMBER cFF performed at 300 K to improve the relaxation of the systems using the fDynamo library.^68–71^ Then, QM/MM PESs using ωB97X-D/AMBER cFF were computed using Gaussian09 ^72^ combined with fDynamo with a dual level strategy^73^ to inspect all the possible reaction mechanisms. Five different mechanisms were proposed and explored, as summarized in Figure 2. More details are provided in the .

**Figure 2:**
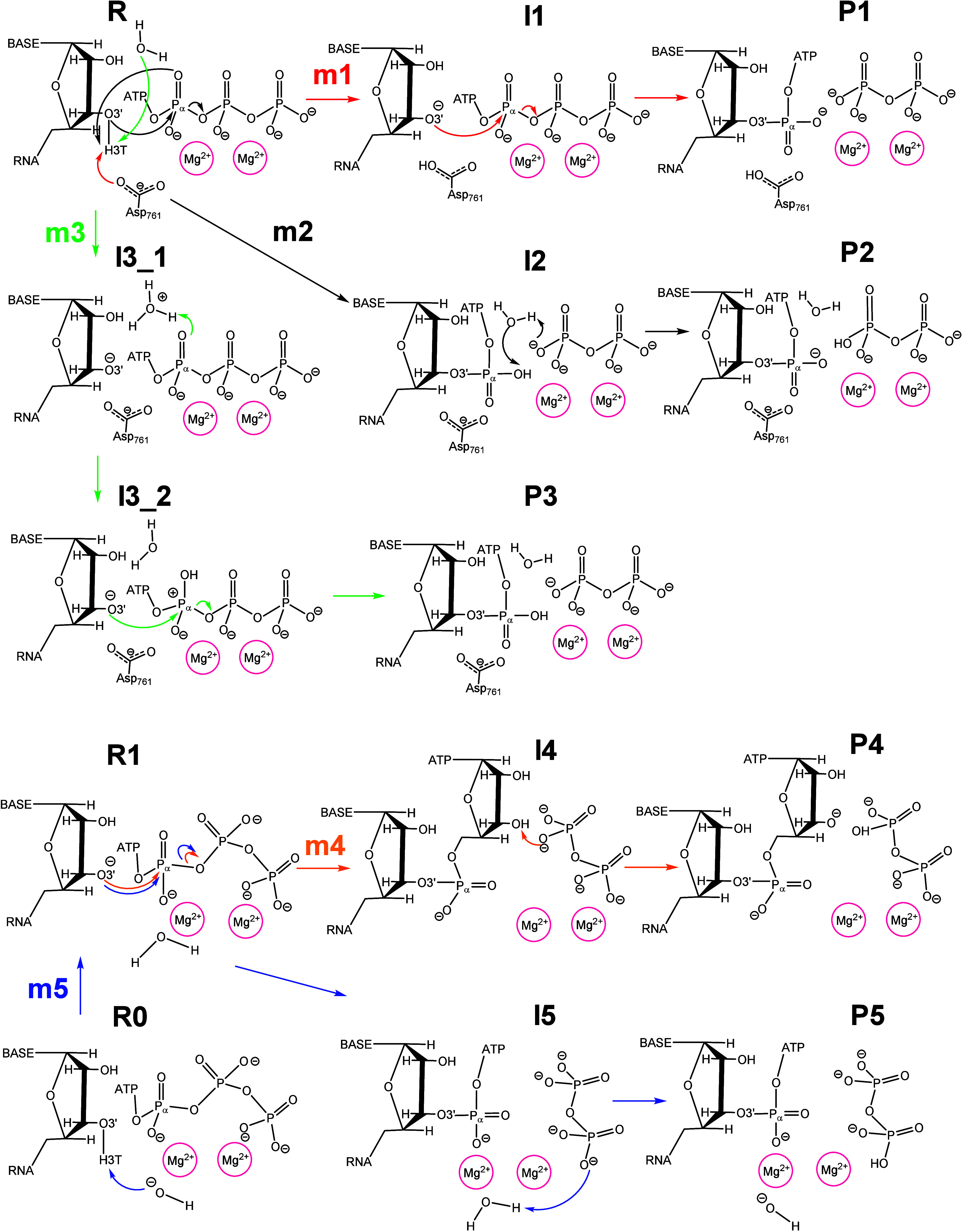
Explored reaction mechanisms for the SARS-CoV-2 RNA-dependent RNA polymerase (RdRp), as derived from the ωB97X-D/AMBER calculations: Mechanism 1 (m1 in red arrows), Mechanism 2 (m2 in black arrows), Mechanism 3 (m3 in green arrows), Mechanism 4 (m4 in orange arrows), and Mechanism 5 (m5 in blue arrows).

All the stationary points were optimized at the ωB97X-D/AMBER level, using Baker’s algorithm.^74^ For all TSs, the Hessian was computed and diagonalized to confirm the existence of a single negative eigenvalue. The convergence of these states was achieved when a 1.0 kJ mol^–1^ Å^–1^ energy gradient was reached. The intrinsic reaction coordinate (IRC)^75,76^ method was applied to the optimized TSs to trace down the minimum energy paths to their corresponding minima. The final structures obtained from IRC were localized and characterized using the same optimization procedure described above. The Hessian was computed and diagonalized to confirm that all eigenvalues are positive. The generated geometries along the IRC were used to calculate the free energy profile of each step of the reaction at the ωB97X-D/AMBER level using FEP method.^77,78^ This consists of sampling the MM region through the path traced by the previously calculated IRC, as well as optimization trajectories until reaching the optimized minima. The change in the MM part during this exploration provokes a polarization in the QM wave function, which allows the exploration of each step at the ωB97X-D/AMBER level with def2-SVP basis set. Thus, the MM environment has a profound impact on the QM/MM potential energy profile.

The structures used in FEP calculations were extracted from the traced IRC and optimization protocol and are characterized by a single *s* coordinate as defined in eq 1,

1 $$s_{i}=s_{i-1}+{\left\{\underset{j\in QM}{\sum }m_{i}\left[{\left(x_{j,i}-x_{j,i-1}\right)}^{2}+{\left(y_{j,i}-y_{j,i-1}\right)}^{2}+{\left(z_{j,i}-z_{j,i-1}\right)}^{2}\right]\right\}}^{1/2}$$

where *m*_*i*_ is the total mass of the QM region and *x*_*j*,*i*_, *y*_*j*,*i*_, and *z*_*j*,*i*_ are the coordinates of the atom *j* of structure *i* of the QM region. According to the *s* coordinate definition, the change of the free energy is computed following eq 2,

2 $$\Delta G_{\mathrm{FEP}}\left(s^{R}-s^{j}\right)=\left[E_{\mathrm{QM}}^{0}\left(s^{j}\right)-E_{\mathrm{QM}}^{0}\left(s^{R}\right)\right]+\left[\mathrm{ZPE}\left(s^{j}\right)-\mathrm{ZPE}\left(s^{R}\right)\right]-k_{B}T\overset{j}{\underset{i=R}{\sum }}\ln\exp{\left[\frac{E_{\mathrm{QM}/\mathrm{MM}}\left(s^{i+1}\right)-E_{\mathrm{QM}/\mathrm{MM}}\left(s^{i}\right)}{k_{B}T}\right]}_{\mathrm{MM},i}$$

where *E*_QM_^0^ is the gas-phase energy of the QM subsystem computed at ωB97X-D level, ZPE is the zero-point energy, *k*_B_ is the Boltzmann constant, and *T* is the temperature. The QM/MM interaction contribution to the free energy difference between two different values of *s* coordinate is obtained by averaging the QM/MM interaction energy (including the polarization energy) over all the MM coordinates of the system explored during the MD simulations obtained for a particular value of the *s* coordinate. In this particular case, 200 ps of QM/MM MD simulation at the ωB97X-D/AMBER level was done for each window at 300 K using the NVT ensemble, maintaining the position of atoms from the QM region fixed during the simulation. Depending on the reaction mechanism explored, the number of windows was different. More details of the FEP calculations are given in the Supporting Information.

The coordinates of all stationary points for the most favorable mechanism (Mechanism 5), as obtained via the QM/MM cFF calculations, were utilized for subsequent QM/MM calculations with the AMOEBA pFF^79^ via the LICHEM^80,81^ (Layered Interacting CHEmical Models) program, to interface Gaussian16 and TINKER 7.0.^82,83^ The ωB97X-D/def2-SVP level of theory and AMOEBAbio18 ^84^ FF were employed for the QM region and the MM environment, respectively. Gaussian16, GDMA 2.3,^85^ and TINKER 7.0 internal modules, POLEDIT and VALENCE were used for the parametrization of the nonstandard residues. To assess the accuracy of the FF and evaluate whether a larger QM region can be effectively replaced by the MM region while maintaining reliable QM/MM energies, two systems with different QM region sizes were studied: one with 102 QM atoms and 6 pseudobond atoms, consistent with the QM/MM MD simulations using the AMBER cFF, and the other with 248 QM atoms plus 6 pseudobond atoms (see Figure S15 of the Supporting Information). The pseudobond approach^86^ was used to treat the covalent boundaries between the QM subsystem and the active MM region, which includes the remaining residues and all solvent molecules within a 25 Å radius from the active site center (MG1472). The noncovalent index (NCI) analysis was performed using the promolecular density method,^87^ and the electron localization function (ELF) analysis^88^ was conducted via basin analysis^89,90^ —both implemented in the Multiwfn V.3.8 program.^91^ All wave functions were derived from stationary points optimized through QM/MM cFF and pFF simulations.

## 3 Results and Discussion

As discussed in the Introduction, the reaction mechanism catalyzed by SARS-CoV-2 RdRp remains under debate. It is generally accepted that it involves a nucleophilic attack by the O3′ atom of the terminal RNA nucleotide on the Pα atom of the incoming nucleotide, with prior deprotonation of the O3′ atom. One key issue, however, is identifying the residue responsible for abstracting the proton. Thus, five plausible mechanisms have been explored in the present study to clarify the enzymatic reaction process (see Figure 2). From the analysis of the MD simulations at the reactant state, R state in Figure 2, alternative residues were selected that could act as a general base that deprotonates the primer O3′ atom.

The first proposed mechanism (Mechanism 1, see Figure 2) involves the abstraction of the proton from the 3′–OH group of the terminal nucleotide by Asp761, followed by a nucleophilic attack on the Pα atom of the incoming ATP nucleotide, which extends the RNA strand by one nucleotide and releases the pyrophosphate moiety. Figure S4 in the Supporting Information shows the 2D-PES for Mechanism 1 (ωB97X-D/AMBER), which shows a TS of the rate-determining step (TS^I1→P1^) 35.8 kcal mol^–1^ above reactants, indicating the mechanism is not energetically feasible. In the second mechanism (Mechanism 2, see Figure 2), the α-phosphate of the incoming ATP acts as a general base, abstracting the proton from the 3′–OH group of the terminal nucleotide in a concerted manner, while the primer O3′ atom nucleophilically attacks the Pα atom. This is followed by proton transfer from the α-phosphate to the β-phosphate occurs via a water molecule. Figure S8 in the Supporting Information shows the FEP free energy profile of both steps for Mechanism 2 at the ωB97X-D/AMBER level. Step 1 is rate-determining with a barrier of 22.8 kcal mol^–1^, much higher than experimental and literature data and other studies, indicating that this mechanism is not viable. Alternatively, with Mechanism 2 in mind, the possible proton transfer from the terminal 3′-OH group to the α-phosphate of the incoming ATP mediated by a water molecule was studied; Mechanism 3 in Figure 2. The 2D potential energy surface (PES) for the initial steps of Mechanism 3, computed at the ωB97X-D/AMBER level, is illustrated in Figure S10 in the Supporting Information. This analysis reveals a stepwise reaction with a significant energy barrier of 28.6 kcal·mol^–1^ for the first step, the proton transfer from the terminal 3′-OH group to the α-phosphate of the incoming ATP mediated by a water molecule, rendering this mechanism energetically unfeasible.

Similar to the mechanism suggested by Orozco and colleagues,^39^ a fourth mechanism (Mechanism 4, see Figure 2) was explored in which the terminal nucleotide’s 3′-OH group was already deprotonated. To start with this mechanism, according to several literature reports, some phosphate groups of the incoming nucleotide are not coordinated to Mg^2+^ ions.^36,39^ Therefore, to prepare a structure where these interactions were absent, we performed preliminary minimizations and short QM/MM MD simulations, using the AM1d method to describe the QM region, to obtain an adequate structure of the reactant state R1 of Mechanism 4 (see Figure 2). This mechanism occurs in two steps: in the first step, the O3′ atom of the terminal RNA nucleotide attacks the Pα atom of the incoming nucleotide, while in the second step, proton transfer occurs from the terminal 3′-OH group of the newly incorporated terminal nucleotide to the β-phosphate group of the previously formed pyrophosphate leaving group. According to the computational analysis of the PESs for both steps, computed at the ωB97X-D/AMBER level and depicted in Figure S12 in the Supporting Information, although the first transition state was successfully located, the structures for the second transition state (TS^I4→P4^, see Figure 2) and the product (P4, see Figure 2) were unstable and could not be characterized, leading us to discard this pathway.”

Upon examining the literature, we found that the high-resolution X-ray structure of the SARS-CoV-2 RNA-dependent RNA polymerase (PDB code: 7BV2) reveals water molecules coordinated with Mg^2+^ ions and positioned between phosphate groups, suggesting that they may contribute to catalysis or stabilization of the active site structure. Additionally, as discussed in the Introduction, previous computational studies have suggested a feasible proton transfer from the 3′–OH group of the terminal nucleotide of the RNA strand to a metal-coordinated or nearby hydroxide ion.^28–34^ Therefore, based on the analysis of the R1 structure in Mechanism 4 (see Figure 2), together with previous experimental and computational evidence, Mechanism 5 was proposed, in which a Mg^2+^-coordinated hydroxide ion acts as a base to abstract the proton from the 3′-OH of the terminal nucleotide. Mechanism 5 was studied starting from reactant state R0 (see Figure 2) with a hydroxide ion coordinated with the Mg^2+^ ion and hydrogen-bonded to the 3′-OH of the terminal nucleotide. The presence of a hydroxide ion bound within an already highly negative charged active site (ca. – 3 au) might seem unexpected. However, the presence of a Mg^2+^ cation and positively charged residues such as Arg555, as well as polar residues like Tyr619, helps stabilize the hydroxide ion. The reactant state R0 was prepared, as in the previous R1, running some minimizations followed by short QM/MM MD simulations using AM1d Hamiltonian to describe the QM region. Thus, a three-step mechanism (Mechanism 5, see Figure 2) was investigated by the exploration of the PES and obtaining the free energy landscape by means of the FEP method at the ωB97X-D/AMBER level. The first step consists of the proton transfer from the 3′–OH group of the terminal nucleotide to a hydroxide group coordinated with the Mg^2+^ ion. This is followed by the nucleophilic attack of the O3′ atom on the Pα atom of the incoming ATP. The final step involves the transfer of a proton from the water molecule formed in the initial step to the γ-phosphate group of the pyrophosphate leaving group. In this mechanism, the hydroxide coordinated with the Mg^2+^ ion is regenerated after the reaction and can subsequently act as a base to deprotonate the next incoming nucleotide. This final mechanism proves to be the most favorable after examining the free energy surfaces of all five mechanisms. In this mechanism, the role of the well conserved Asp761 residue in RNA polymerases, which coordinates one of the Mg^2+^ ions, is to stabilize the position and geometry of Mg^2+^ ion, which in turn facilitates the deprotonation of the water molecule coordinated with the metal giving rise the formation of the hydroxide ion to act as a base. Detailed descriptions of all studied mechanisms are available in the Supporting Information.

The PES of all the mechanisms computed at the ωB97X-D/AMBER (cFF) level are shown in Figures 3A and 3B. As previously described, Mechanisms 1, 2, and 3 were energetically unfeasible (see Figure 3A). Mechanism 4 was discarded because both TS^I4→P4^ (see Figure 2) and the product P4 (see Figure 2) were unstable and could not be characterized, whereas Mechanism 5 is the most plausible pathway (see Figure 3B). The free energy landscape of Mechanism 5, FEL, computed via the FEP method at the ωB97X-D/AMBER (cFF) level, as described in Methods, is shown in Figure 3C, while the optimized ωB97X-D/AMBER (cFF) TS structures are displayed in Figure 4. More detailed information on the exploration of the PESs and the subsequent free energy profile calculations are given in the Supporting Information. According to the results, the rate-determining step corresponds to the second step, the nucleophilic attack of the O3′ atom of the terminal nucleotide of the RNA strand to the Pα atom of ATP, with a free energy barrier of 15.2 kcal mol^–1^. The free energy barriers of the first and third steps are 4.8 and 3.7 kcal mol^–1^, respectively. From the thermodynamic point of view, the reaction is exergonic with a reaction free energy of 2.1 kcal mol^–1^. Based on these results, this reaction mechanism looks plausible to explain the reaction process catalyzed by the RdRp from SARS-CoV-2.

**Figure 3:**
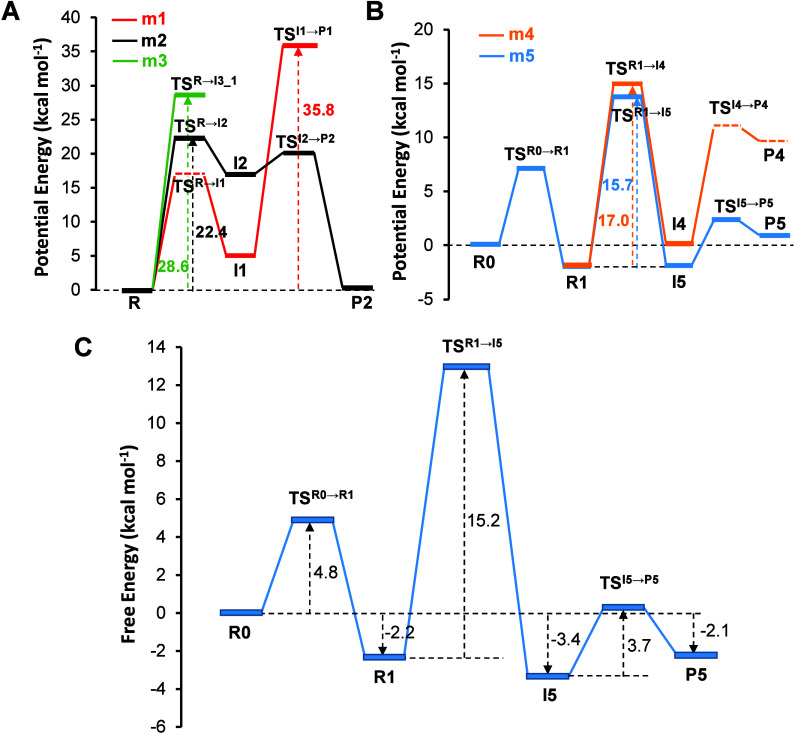
(A) ωB97X-D/AMBER cFF PES for mechanisms m1, m2, and m3, (B) ωB97X-D/AMBER cFF PES for mechanisms m4 and m5, and (C) ωB97X-D/AMBER cFF free energy landscape (FEL) of the most plausible mechanism (m5). Energy values are in kcal mol^–1^.

**Figure 4:**
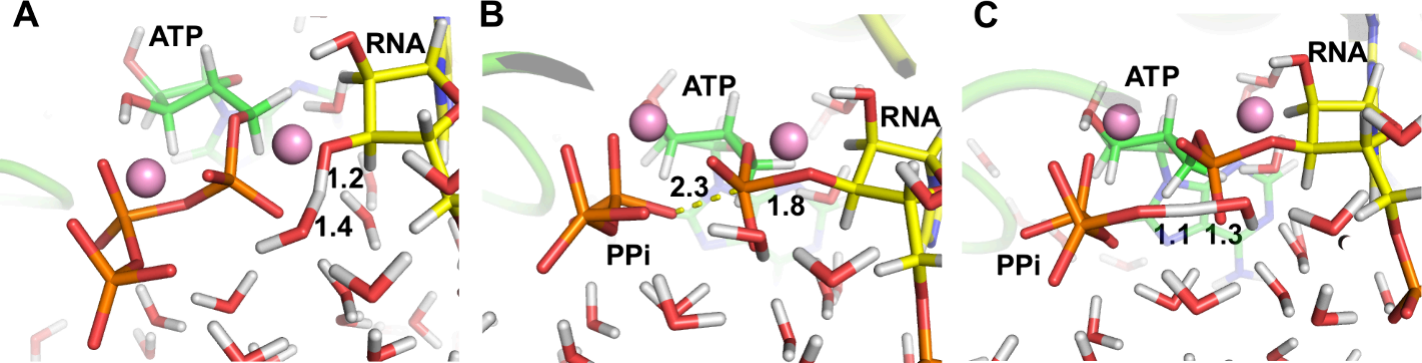
ωB97X-D/AMBER cFF optimized transition state structures for (A) step 1 ((TS^R0→R1^)), (B) step 2 (TS^R1→I5^), and (C) step 3 (TS^I5→P5^). The carbon atoms of the incoming nucleotide (ATP) and the 3′-terminal nucleotide of the RNA strand (RNA) are shown in green and yellow, respectively. The Mg^2+^ ions are shown in pink. The bond-breaking and bond-forming distances are in Å. The PPi label denotes the formed pyrophosphate leaving group.

We used the optimized coordinates of all the stationary points in Mechanism 5 for subsequent QM/MM calculations with the AMOEBA polarizable force field (QM/MM pFF). Two model systems featuring different QM region sizes (102 and 248 atoms) were examined to evaluate whether substituting the larger QM region with an MM region still yields reliable QM/MM PES values.

Consistent with the QM/MM cFF FEL in Figure 3C, the QM/MM cFF PES of the three steps in Mechanism 5 (see Figure 3B and Figure S14) also indicate that the rate-determining step is the second step, i.e., R1 → TS^R1→I5^, with similar energetic values. Table 1 presents the relative potential energies comparing the QM/MM cFF and pFF approaches, while a graphical comparison is shown in Figure S16. The QM/MM pFF potential energy barrier of the rate-limiting step with the smaller QM region is 15.2 kcal mol^–1^, which is slightly smaller than the values obtained from the large QM region and the previous PES with QM/MM cFF (16.2 and 15.7 kcal mol^–1^, respectively). The potential energy barriers for the other two steps (R0 → TS^R0→R1^ and I5 → TS^I5→P5^) are comparable across the tested approaches. However, a closer examination of the overall energetics reveals that the polarizable QM/MM approach with a smaller QM region produces results that agree as well with the reference values as those obtained using the larger QM model. This comparable performance may be attributed to the fact that, for the system studied, the key features for reactivity are adequately captured within the smaller QM region, while the polarizable MM potential effectively accounts for the environmental effects, and together provide an appropriate representation of the overall energetics of the reactive process. The entire reaction in both models was suggested to be exergonic with a reaction potential energy of −1.7 kcal mol^–1^, comparable to the reaction free energy of −2.1 kcal mol^–1^ obtained by QM/MM MD calculations with cFF.

**Table 1:** Relative QM/MM Energies for Mechanism 5, Optimized with QM/MM cFF and QM/MM pFF^a^

	relative energies (kcal mol^–1^)					
QM/MM method	R0 → TS^R0→R1^	R0 → R1	R1 → TS^R1→I5^	R1 → I5	I5 → TS^I5→P5^	I5 → P5
QM/MM cFF free energies^a^	4.8	–2.2	15.2	–1.2	3.7	1.3
QM/MM cFF^b^	7.1	–1.9	15.7	0.2	4.2	3.2
QM/MM pFF^c^ (102 atoms)	7.9	–2.5	15.2	–1.7	4.5	2.5
QM/MM pFF (248 atoms)	5.7	–3.6	16.2	–2.8	4.4	2.5

RMSD^d^ (Å)	R0	TS^R0→R1^	R1	TS^R1→I5^	I5	TS^I5→P5^	P5
MM region	0.18	0.17	0.13	0.15	0.14	0.13	0.13
QM region	0.05	0.22	0.11	0.16	0.08	0.05	0.04

a The table also includes RMSD values comparing the QM and active MM regions of all stationary points between the two approaches.

b Free energy values were obtained from the FEL diagram in Figure 3C.

c Fixed-charged potential energy (QM/MM cFF) values were obtained from the PES diagram in Figure S14.

d Polarizable potential energy (QM/MM pFF) values were obtained from Figure S16.

e RMSD values reflect the differences between QM/MM cFF and QM/MM pFF (102 atoms), given the close similarity between models with smaller and larger QM regions.

The RMSD values between the cFF QM/MM and pFF QM/MM (102 atoms) structures were also calculated to assess the structural agreement between the two methods (see Table 1). For the MM region, RMSD values ranged from 0.13 to 0.18 Å across all stationary points, indicating excellent structural consistency. Similarly, the QM region exhibited RMSD values between 0.04 and 0.22 Å, with slightly higher deviations observed for transition states (e.g., TS^R0→R1^ and TS^R1→I5^). These low RMSD values indicate that both the fixed-charge and polarizable QM/MM representations yield nearly identical geometries, demonstrating that the smaller QM region in the polarizable model preserves the key structural features observed across methods. The close similarity between the models, particularly for the reactant (R0) and product (P5) states, further supports the reliability of using a reduced QM region in polarizable QM/MM calculations without significant loss of accuracy.

The NCI analysis of Mechanism 5 (illustrated in Figure 5), highlights the interplay of attractive and repulsive noncovalent interactions along the reaction coordinate. In the reactant state (R0), prominent blue surfaces between the terminal nucleotide’s 3′–OH group and the hydroxide group coordinated with the Mg^2+^ ion highlight strong stabilizing interactions, which facilitate the initiation of the deprotonation reaction and the production of H_2_O. As the system progresses to the reactant complex (R), green and blue surfaces emerge between the terminal nucleotide’s negatively charged O3′ atom and the Pα atom of the incoming ATP, indicating favorable interactions that stabilize the prereaction state.

**Figure 5:**
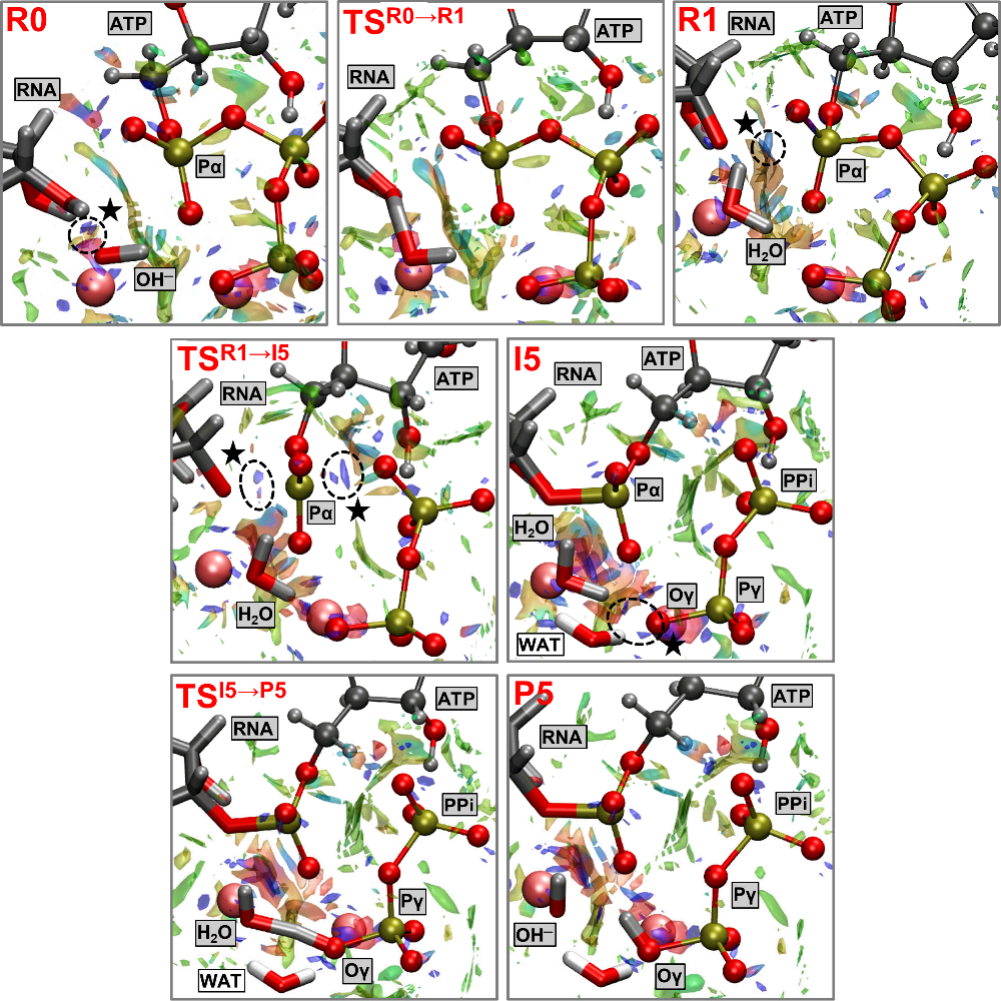
Noncovalent interaction (NCI) surfaces surrounding the active site residues in critical structures along the polymerase reaction (Mechanism 5). The surfaces are color-coded on a RGB scale; Key: green and blue represent strong and weak interactions (e.g., hydrogen bonds and van der Waals forces, respectively), while red indicates repulsive interactions. Visualization was performed using an isovalue of 0.4 au and a color scale of −0.05 au < sign­(λ_2_)­ρ < 0.05 au. A cubic grid of 200 au with an isovalue of 0.8 au and a high-quality grid spacing of 0.10 Bohr was used for the basin illustration. Surfaces of particular interest are highlighted with dotted circles and marked with a solid star (★) for clarity. NCI surfaces were generated from wave functions obtained using the QM/MM pFF model with a 102-atom QM region, due to its close similarity with the results from the 248-atom QM/MM pFF calculations.

As the system approaches the rate-limiting transition state (TS^R1→I5^), the appearance of mixed blue surfaces encircled by red regions, revealing both attractive and repulsive interactions, reflect the buildup of electronic and steric strain as the catalytic groups approach one another. This strain is critical for driving bond reorganization, as it underscores the system’s need to overcome energetic barriers during bond formation and cleavage.

In the subsequent intermediate state (I5), the reemergence of blue and green regions signifies the reestablishment of stabilizing interactions of the newly formed bonds and the formation of inorganic pyrophosphate (PPi). Notably, blue surfaces between the Oγ atom of PPi and two water molecules (H_2_O and WAT in Figure 5) highlight strong interactions on the NCI scale, which facilitate the second deprotonation reaction, leading to the production of OH^–^ and the protonated PPi.

Finally, in the product state (P5), the dominance of green surfaces suggests that the system has reached an energetically favorable configuration, consistent with the formation of a stable phosphodiester bond. Overall, these NCI surfaces provide a detailed depiction of the delicate balance between stabilizing and destabilizing forces that drive the catalyzed reaction in Mechanism 5.

Electron localization function, ELF, analysis can provide a detailed picture of the electronic rearrangements occurring during the RdRp-catalyzed polymerization reaction in Mechanism 5. Examination of the ELF basins reveals the redistribution of electron density during bond breaking and formation processes.^92,93^ Calculated ELF basins obtained from the pFF QM/MM and cFF QM/MM wave functions, as shown in Figure 6A (detailed results in Table S1, Supporting Information), suggest the formation of trisynaptic basins (O,Mg1,H3′), (O3′,Pα,O3α), and (Pγ,O3γ,H) in TS^R0→R1^, TS^R1→I5^, and TS^I5→P5^, respectively.

**Figure 6:**
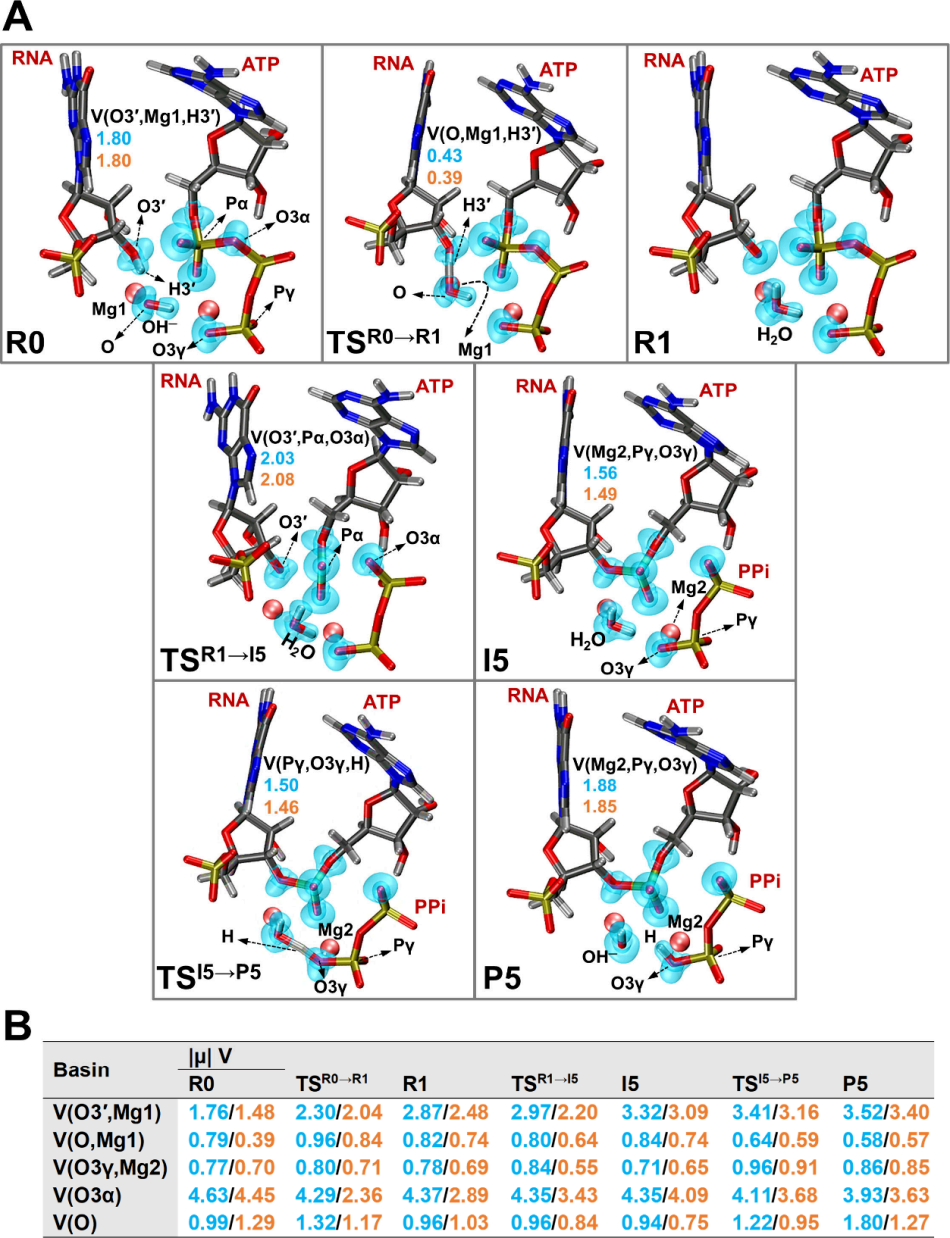
(A) ELF basins (isovalue = 0.8 au) along with their corresponding electron populations (*e* ^*–*^) for key structures along the polymerization reaction in Mechanism 5. (B) Dipole moment magnitudes (|μ| V in au) for some of the basins involved in the catalytic reaction. Key: Monosynaptic basins (e.g., V­(X)) represent lone pairs or nonbonding regions, disynaptic basins (e.g., V­(X,Y)) correspond to covalent bonds between two atoms, and trisynaptic basins (e.g., V­(X,Y,Z)) indicate bonding regions involving three atoms, such as in delocalized or multicenter bonds. Light blue values are derived from QM/MM pFF optimized structures at ωB97X-D/def2-SVP//AMOEBAbio18, while orange values are calculated from QM/MM cFF wave functions at ωB97X-D/def2-SVP//AMBER. The dipole moment difference discussed in the main text reflects the discrepancy between these two models. ELF basins and associated properties were computed from wave functions obtained using the QM/MM pFF model with a 102-atom QM region, due to its close similarity with the results from the 248-atom QM/MM pFF calculations.

In the reactant state (R0), a trisynaptic basin forms comprising the 3′-OH group of the terminal nucleotide and the catalytic Mg^2+^ ion (V­(O3′,Mg1,H3′) = 1.80 *e* ^*–*^). The presence of this trisynaptic basin in the reactant suggests a potential facilitating role of the catalytic Mg in initiating the first deprotonation reaction. Furthermore, the basin corresponding to the interaction between the terminal nucleotide’s O3′ and the catalytic Mg^2+^ ion (O3′,Mg1) exhibits relatively stable populations in the early stages, with fluctuations emerging as the reaction progresses (see V­(RNA^O3′^,Mg1) in Table S1, Supporting Information). This behavior also underscores the critical role of the metal ion in stabilizing key intermediates and transition states throughout the reaction.

A trisynaptic basin also begins to form in TS^R1→I5^ and persists up to the final product (P5), involving the γ-phosphate group of the incoming ATP nucleotide and the second Mg^2+^ ion (V­(Mg2,ATP^Pγ^,ATP^O3γ^) in Table S1, Supporting Information). The formation of this trisynaptic basin, along with its tangible electron population progressing from I5 to P5 along the reaction pathway (see V­(Mg2,Pγ,O3γ) in Figure 6A), further supports a charge-stabilizing role of the second metal ion during the nucleophilic attack step of the reaction.

Similarly, basins associated with the nucleophilic attack, such as (RNA^O3′^,ATP^Pα^) and (ATP^Pα^,ATP^O3α^) in Table S1, exhibit slight but consistent changes in electron populations, reflecting the gradual formation of a new bond between the primer nucleotide and the incoming phosphate moiety of ATP. Additionally, the evolution of ELF basins and the observed shifts in electron populations of the cleaved pyrophosphate’s O3α (V­(ATP^O3α^,ATP^Pβ^) and V­(ATP^O3α^) in Table S1, Supporting Information) mirrors the progression of the reaction from substrate activation to the formation of a stable phosphodiester bond.

As noted above, the RMSD values indicate that the orientations of entities in the QM region and active MM environment are essentially unchanged regardless of whether pFF or cFF is applied. To further elucidate these effects, we computed dipole moments of the ELF basins to assess whether changes in charge distribution can account for the observed differences.

An extensive list of dipole moments of the ELF basins (|μ| V) for the key structures in Mechanism 5 is provided in Table S2 of the Supporting Information. As summarized in Figure 6B, the (O3′,Mg1) basin shows an increasing dipole moment as the reaction progresses from R0 to P5, reflecting the growing influence of Mg^2+^ in stabilizing negative charge on the terminal nucleotide’s O3′. In particular, the (O3′,Mg1) and (O,Mg1) dysinaptic basins, which involve the catalytic Mg^2+^ ions, exhibit larger dipole moments in the pFF QM/MM calculations compared to the cFF QM/MM results. These differences are most pronounced in R0 for the (O,Mg1) basin and in R1 and TS^R1→I5^ for the (O3′,Mg1) basin, aligning with the electron population data and the reaction direction. This observation indicates that explicit polarization effects allow for a more pronounced redistribution of electron density around these critical regions, ultimately stabilizing partial charges that develop during bond cleavage and formation.

The stepwise changes in dipole moments across the reaction coordinate align with the trends in electron population shifts noted in the ELF analysis. Basins implicated in the nucleophilic attack and PPi formation—such as (O3γ,Mg2) and (O3α) in Figure 6B—show elevated dipole moment differences as the reaction proceeds from R0 to TS^R1→I5^, reflecting the local buildup of negative charge and its subsequent stabilization by the metal ions and nearby residues in polarizable QM/MM calculations compared to cFF QM/MM. These correlated shifts in population and dipole moments highlight the role of polarization in more effectively capturing the balance of electrostatic forces within the active site.

A different trend is observed for the dipole moment values associated with the hydroxide oxygen monosynaptic basins, |μ| V­(O). The hydroxide oxygen coordinated with Mg1 experiences a less polarized environment in both R0 and R1 in our polarizable QM/MM calculations (see Figure 6B). This reduction in polarization may be attributed to the stabilizing effect of Mg1, as discussed earlier. In contrast, the hydroxide group experiences a significantly more polarized environment in our polarizable QM/MM calculations during the final step (I5 → P5), which aligns with the nature and direction of the deprotonation reaction from the OH^–^ to PPi.

Overall, the dipole moment results support the conclusion that polarizable QM/MM models can provide a more nuanced depiction of the electronic environment. By accounting for polarization, these models produce energetics that can better align with free energy profiles.

### Comparison with Previous Experimental and Theoretical Studies on the Mechanism of RNA Polymerases

Upon comparing our results with previous mechanistic studies of SARS-CoV-2 RdRp, differences can be observed in the mechanism, structures, and energetics. Concerning the mechanism, the most feasible mechanism proposed in our study is similar to others proposed for RNA Pol II, where a bulk OH^–^ ion, hydrogen bonded to the terminal RNA and near the Mg^2+^ ion, is the base responsible for abstracting the proton from the 3′-OH group of the terminal nucleotide.^31–34^ In our study, the OH^–^ molecule is coordinated with an Mg^2+^ ion, as previously proposed for DNA polymerases.^28–30^ In contrast, in the work by Aranda et al. on SARS-CoV-2 RdRp, the deprotonation of the O3′ was proposed to be done by the pyrophosphate leaving group resulting from the previous nucleotide incorporation.^39^ We examined this reaction as a plausible mechanism (Mechanism 4, see Figure 2), but it was not possible to localize and characterize the transition state and the product of this proton transfer step. Consequently, the reaction mechanism proposed by Aranda et al. was dismissed.

The nucleophilic attack step is the common step proposed in various studies of RNA polymerases.^31,32,36,39^ However, no consensus has been reached regarding the final step—the protonation of the pyrophosphate leaving group, which is considered essential for its release. In our investigation, the water molecule produced in the first step and coordinated with an Mg^2+^ ion, is the responsible for transferring a proton to one oxygen atom of the γ-phosphate of the pyrophosphate leaving group, thereby regenerating the OH^–^ molecule in the active site to deprotonate the next incoming nucleotide. In contrast, Bignon and Monari proposed that a lysine residue is responsible for protonating the pyrophosphate-leaving group in the SARS-CoV-2 RdRp.^36^

Regarding the structures, our results show that not all the phosphates of the incoming nucleotide to be bound to the RNA strand are coordinated with the Mg^2+^ ions. In particular, the β-phosphate was not coordinated with the Mg^2+^ ions, in agreement with the study by Bignon and Monari.^36^ In contrast, the γ-phosphate was the one not coordinated with the Mg^2+^ ions in the study of Aranda et al.^39^ When comparing the transition state structure of the rate-limiting step, the nucleophilic attack of the O3′ atom of the terminal nucleotide of the RNA strand to the Pα atom of ATP, the bond-breaking distance is larger than the bond forming distance in the localized structure (depicted in Figure 4B). This structure is similar to the transition state structure obtained by Aranda et al. for the RdRp with ATP, where the bond-breaking distance is slightly larger than the bond-forming distance.^39^ However, it differs from the transition state described by Bignon and Monari, who, despite claiming that the phosphate group is equidistant from the donor and acceptor oxygen atoms, its location on the 2D free energy surface characterized a structure with a bond-breaking distance significantly shorter than the bond-forming distance. This suggests that their transition state structure is closer to the reactants.^36^ Notably, they studied the reaction mechanism of the SARS-CoV-2 RdRp using GTP as the incoming nucleotide.^36^

Regarding the energetics of the reaction, the activation free energy in our study is 15.2 kcal mol^–1^, which is similar to the value reported by Aranda et al. (16.2 kcal mol^–1^)^39^ but larger than the value reported by Bignon and Monari (10 kcal mol^–1^).^36^ From the thermodynamic perspective, the rate-limiting step is exergonic, yielding energy of – 1.2 kcal mol^–1^, which is closer to the value obtained by Aranda et al. (approximately −5 kcal mol^–1^),^39^ while the reaction free energy reported by Bignon and Monari is much more negative (−18 kcal mol^–1^).^36^

## 4 Conclusions

The molecular mechanism of SARS-CoV-2 RNA-dependent RNA polymerase (RdRp) has been explored based on QM/MM simulations with cFF and pFF. The use of different FF allowed addressing technical questions related with the impact of the pFF, in comparison with the partial fixed charges FF, while the generation of the free energy landscape of the chemical steps, obtained by means of FEP method, provided a detailed description of the multistep process involved in nucleotide incorporation to RNA.

Thus, from the mechanistic point of view, a key finding of our study is the detailed elucidation of the deprotonation mechanism of the 3′–OH group of the terminal nucleotide, which is a prerequisite for its nucleophilic attack and, up to now, it was assumed but not explored in previous studies. The present study rigorously investigated five plausible mechanistic pathways, ultimately identifying a highly favorable, three-step process. This mechanism involves an initial proton transfer to an Mg^2+^-coordinated hydroxide group, followed by the crucial nucleophilic attack of the O3′ atom on the α-phosphate of the incoming nucleotide. Finally, the mechanism concludes with a proton transfer from the water molecule coordinated with a Mg^2+^ ion, and obtained at the first step, to the pyrophosphate leaving group, which is considered to be required for its release, regenerating the active site. The identification of the nucleophilic attack as the rate-determining step, with a calculated free energy barrier of 15.2 kcal mol^–1^, supports the plausibility of this reaction mechanism in explaining the process catalyzed by the SARS-CoV-2 RdRp.

From the methodological standpoint, our results demonstrate that, a qualitatively equivalent description of the computed process is obtained with both schemes, albeit with an increase in computational cost when employing pFF. For the present enzymatic system, it is observed that both cFF and pFFs show equivalent structural and energetic results, suggesting that cFF provide an appropriate representation of the environment. However, the application of noncovalent interaction (NCI) and electron localization function (ELF) analyses further enhanced the understanding of electron redistribution and interactions during the catalytic process and indicate that pFFs offer an improved representation of the electronic environment within the enzyme’s active site as observed by the differences in calculated electric fields at selected points.

These results provide a deeper understanding of the SARS-CoV-2 RdRp catalytic mechanism and highlights the importance of advanced computational techniques in deciphering complex biochemical processes. The detailed mechanistic insights and the validation of pFFs provide a robust foundation for future drug design efforts, specifically aiding in the rational design of novel antiviral inhibitors that can effectively target emerging RNA viruses.

## Supplementary Material





## Notes

Additional computational details; Detailed information on Mechanisms 1–5, including PESs and FESs; Results of the ELF populations and dipole moments for the critical structures along the polymerization reaction in Mechanism 5 (PDF)
All the coordinates of stationary point structures optimized at QM/MM cFF and QM/MM pFF level (ZIP)
