# Supplementary material for: Comparing Force Field Treatments in QM/MM Studies of the SARS-CoV‑2 RNA-Dependent RNA Polymerase (RdRp) Mechanism
Source: J Chem Theory Comput. 2025 Nov 24;21(23):12342–55. doi: 10.1021/acs.jctc.5c01399 (PMC12874388; doi:10.1021/acs.jctc.5c01399)
Supplement: Supplementary file 1 [file ct5c01399_si_001.pdf]

Supporting Information

Comparing Force Field Treatments in QM/MM Studies of the SARS-CoV-2 RNA-Dependent RNA Polymerase (RdRp) Mechanism

Maite Roca,[1,#] Yazdan Maghsoud,[2,3,#] G. Andrés Cisneros,[2,4,*] Katarzyna Świderek,[1,*] Vicent Moliner[1,*]

1. BioComp Group. Institute of Advanced Materials (INAM), Universitat Jaume I, Castelló de la Plana 12071, Spain
2. Department of Chemistry and Biochemistry, The University of Texas at Dallas, Richardson, TX 75080, USA
3. Department of Biochemistry and Molecular Pharmacology, Baylor College of Medicine, Houston, TX 77030, USA
4. Department of Physics, The University of Texas at Dallas, Richardson, TX 75080, USA

# These authors contributed equally to this work

Potential Energy Surfaces and Free Energy Profiles

For the study of all the possible reaction mechanisms, quantum mechanics/molecular mechanics (QM/MM) simulations were performed to explore the potential energy surfaces (PES) using Gaussian09[1] combined with fDynamo.[2] The QM region was described using the ωB97X-D[3] method with the def2svp basis set, while the AMBER ff14SB[4] was used to describe the residues of the protein, AMBER force field ff99[5] with the parmbsc0[6] and parmχOL3[7] dihedral modifications, supplemented by van der Waals parameters for phosphates[8] was used for the RNA, ZAFF[9] FF for Zn^{2+} ions, and the TIP3P[10] force field was selected to describe the water molecules and the K^+ ions. Parameters for Mg^{2+} were adapted from Villa and co-workers.[11] The truncated system consists of 1371 amino acid residues, 54 nucleotides, 2 Zn^{2+} ions, 2 Mg^{2+} ions, the ATP molecule, 61 K^+ ions to neutralize the system, and 7402 water molecules (**Figure S1**). All residues positioned more than 20 Å away from the ATP molecule were fixed during the QM/MM optimizations and QM/MM free energy perturbation (FEP) simulations. In all the reaction mechanisms studied, the QM region consists of the ATP (the incoming nucleotide), part of the 3′-terminal nucleotide of the RNA, the Mg^{2+} ions, a water molecule, and the side chains of the residues involved in the coordination spheres of the Mg^{2+} ions (see Figure S2). Once the PES were obtained, the transition state structures were localized and characterized. Afterward, the intrinsic reaction coordinate (IRC) method was applied to the optimized TSs to trace down the minimum energy paths to reactants and product valleys. The final structures obtained from IRC were localized and characterized as minima on the PES. The geometries generated along the IRC of all the feasible reaction mechanisms were used to calculate the free energy profiles of each step of the reaction at the ωB97X-D/AMBER level using free energy perturbation (FEP) methods. All the details of the calculations of all the reactions' mechanisms are given in the following sections.

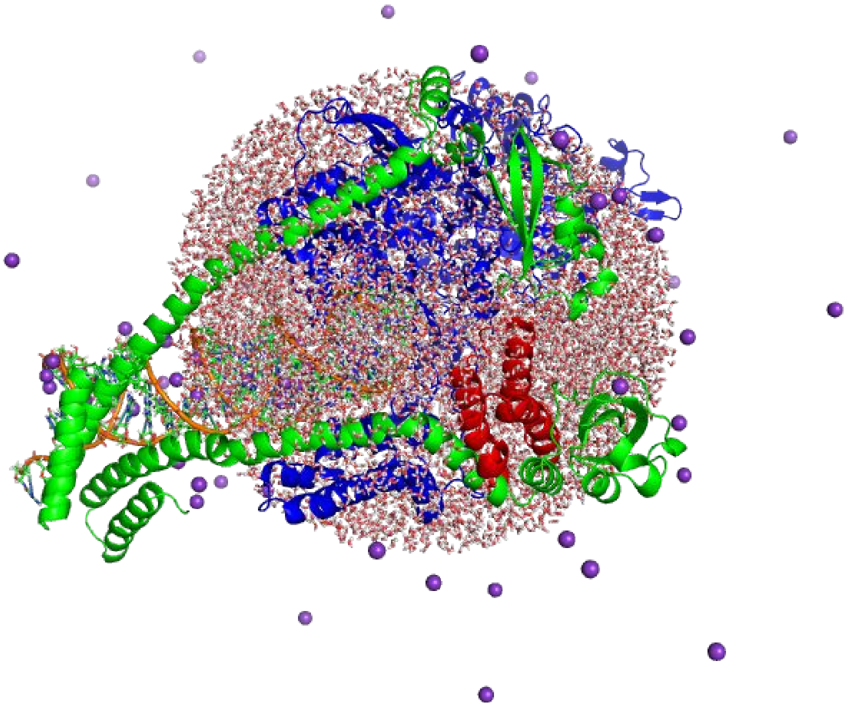

Figure S1. The truncated system obtained by removing all the water molecules located outside a sphere of 40 Å around the ATP residue.

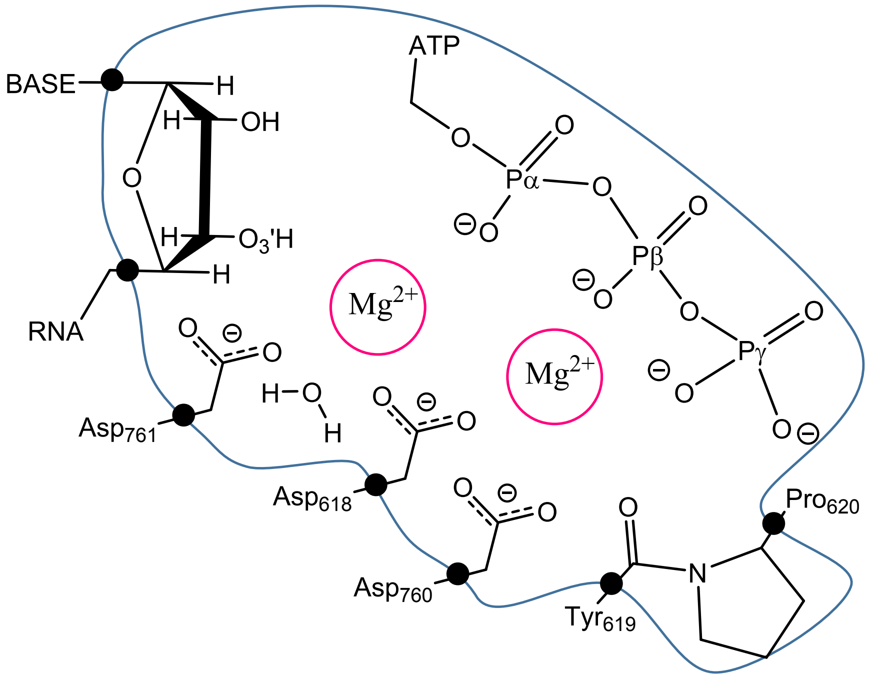

Figure S2. Schematic representation of the region (inside the blue curve) described quantum mechanically for the study of the reaction mechanism catalyzed by RdRp of SARS-CoV-2. The black dots indicate the positions of link atoms.

Mechanism 1. Transfer of the Proton to Asp761 and Nucleophilic Attack

In M*echanism 1,* depicted in **Figure S3** and **Figure 2** in the main text, the Asp761 residue was the base to abstract the proton from the 3′-OH group of the terminal nucleotide, and consecutively, the nucleophilic attack of O3′ atom of the terminal nucleotide of the RNA strand to the Pα atom of the incoming ATP nucleotide takes place. This reaction mechanism is similar to other studies where DNA polymerases are involved.[12-15]

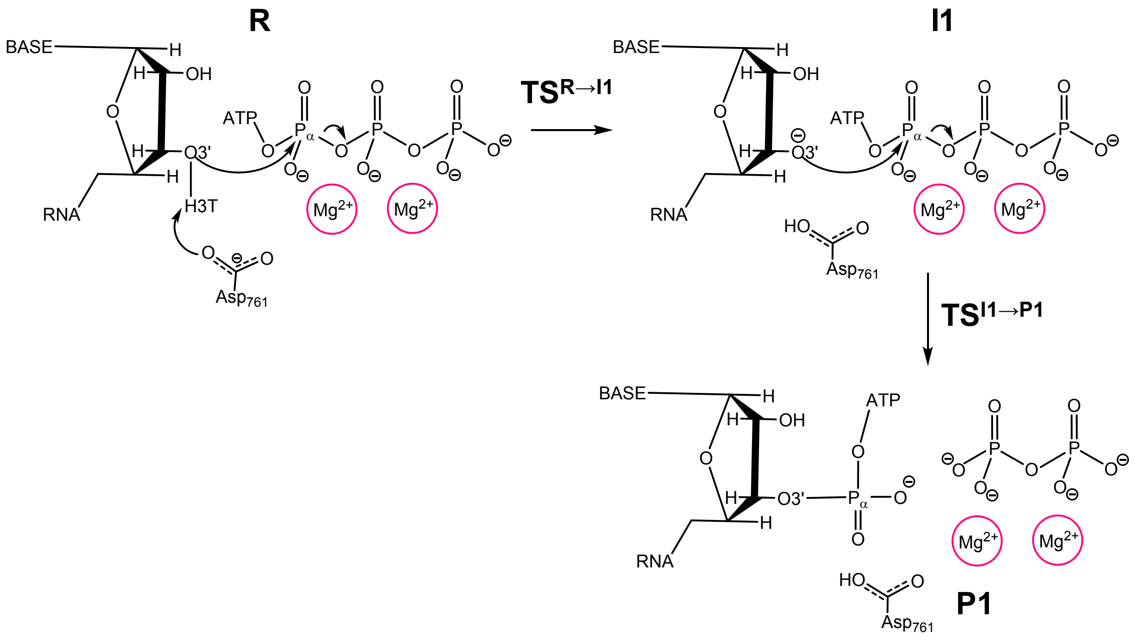

Figure S3. *Mechanism 1*, where the Asp761 residue is the base that abstracts the proton from O3′ of the terminal nucleotide of the RNA strand to form the nucleophile to attack the Pα atom of ATP subsequently. R, TS$^{R\rightarrow I1}$, I, TS$^{I1\rightarrow P1}$, and P1 denote reactant, transition state of step 1, intermediate 1, transition state of step 2, and product 1, respectively.

To explore *Mechanism 1*, a two-dimensional potential energy surface (2D-PES) at the ωB97X-D/AMBER level was performed using as selected distinguished reaction coordinates the antisymmetric combination of the bond breaking and bond forming distances (D(O3′ RNA, H3T RNA) - D(H3T RNA, OD2 Asp761) and D(O3A ATP, PA ATP) – D (PA ATP, O3′ RNA)) and the number of steps was 35 and 40 for each reaction coordinate, respectively. The employed force constant was 5000 kJ mol^{-1} Å^{2}, and the step width was 0.1 Å.

Figure S4 shows the 2D-PES of *Mechanism 1* computed at the ωB97X-D/AMBER level. Some structures around the transition state of step 1 (TS$^{R\rightarrow I1}$) and the transition state of step 2 (TS$^{I1\rightarrow P1}$) regions were selected to localize and characterize the transition state

structures, but $TS^{R \rightarrow I1}$ could not be localized. $TS^{I1 \rightarrow P1}$ was localized and characterized, but its energy was too high compared to the energy of reactants (R) (35.8 kcal mol^{-1}). Thus, this reaction mechanism was deemed not feasible energetically.

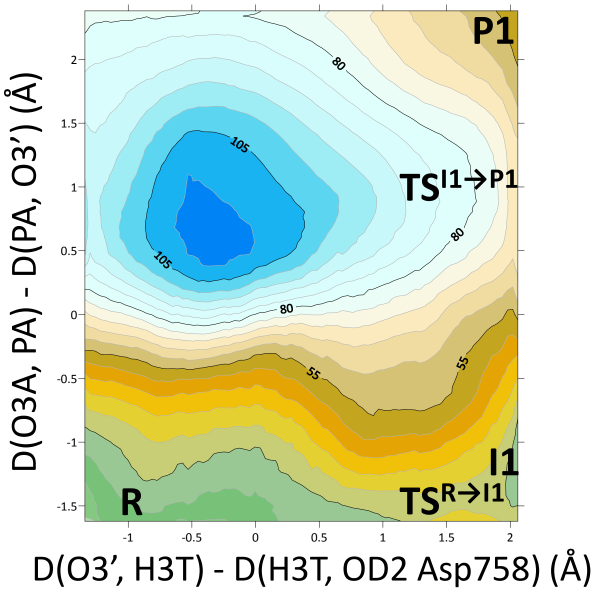

Figure S4. 2D-PES at the ωB97X-D/AMBER level of *Mechanism 1*. Isocontour lines are drawn at each 5 kcal mol^{-1}. R, $TS^{R \rightarrow I1}$, I1, $TS^{I1 \rightarrow P1}$, and P1 denote reactant, transition state of step 1, intermediate 1, transition state of step 2, and product 1, respectively. Energies are in kcal mol^{-1}.

Mechanism 2. Transfer of the Proton to α-phosphate Group Concerted with the Nucleophilic Attack

Mechanism 2 is depicted in **Figure S5** and **Figure 2** in the main text. It consists of the proton transfer from the 3′-OH group of the terminal nucleotide of RNA to an oxygen atom of the α-phosphate of the incoming nucleotide (ATP), concerted with the nucleophilic attack of the O3′ atom to the Pα atom of ATP. Subsequently, the proton transfer from the α-phosphate of ATP to a water molecule and from the water molecule to an oxygen atom of the β-phosphate occurs. This mechanism encompasses not only the deprotonation of the O3′ atom but also the protonation of the pyrophosphate leaving group, which is considered necessary for its release.

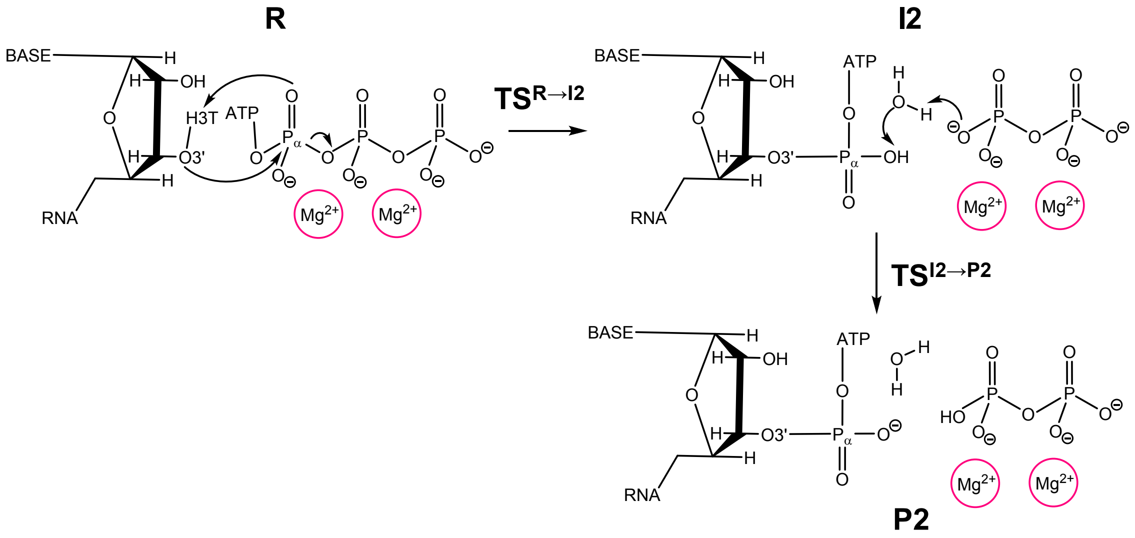

Figure S5. *Mechanism 2* involves proton transfer from the O3′ atom of the terminal nucleotide of the RNA strand to the α-phosphate group, occurring concurrently with the nucleophilic attack on the Pα atom of the incoming nucleotide. Following this, the proton is transferred from the α-phosphate to the β-phosphate via a water molecule. R, $TS^{R \to I2}$, I2, $TS^{I2 \to P}$, and P2 denote reactant, transition state of step 1, intermediate 2, transition state of step 2, and product 2, respectively.

In *Mechanism 2*, the first step was studied using the exploration of a 2D-PES at the ωB97X-D/AMBER level using as selected distinguished reaction coordinates the antisymmetric combination of the bond breaking and bond forming distances (D(O3′ RNA, H3T RNA) - D(H3T RNA, O2A ATP) and D(O3A ATP, PA ATP) – D (PA ATP, O3′ RNA)) and the number of steps was 40 and 30 for each reaction coordinate, respectively. The potential energy surface of the second step was performed using a single distinguished reaction coordinate (D(H3T RNA, O2A ATP) – D(H3T RNA, O WAT)),

and the number of steps was 16. The force constant used for all the reaction coordinates was 5000 kJ mol^{-1}Å^{2}, and the step width was 0.1 Å.

The 2D-PES of the first step of *Mechanism 2* at ωB97X-D/AMBER level is shown in **Figure S6**. TS$^{R\rightarrow I2}$ was localized and characterized at ωB97X-D/AMBER level. From TS$^{R\rightarrow I2}$, the intrinsic reaction coordinate (IRC)[16, 17] method was applied to trace down the minimum energy paths to reactants and product valleys. The final structures obtained from IRC were localized and characterized as minima on the PES, reactants (R), and intermediate (I2). The obtained potential energy barrier is 22.4 kcal mol^{-1}. Figure S7 shows the potential energy profile of step 2 obtained after the localization and characterization of TS$^{I2\rightarrow P2}$ and computing the IRC to get the intermediate (I2) and product (P2). The potential energy barrier of this step corresponds to 3.1 kcal mol^{-1}.

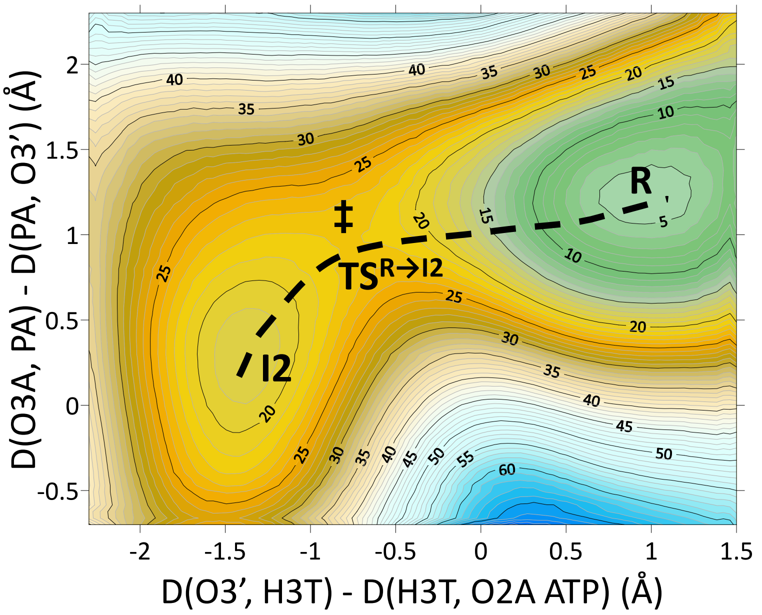

Figure S6. 2D-PES at the ωB97X-D/AMBER level of the first step of *Mechanism 2*. Isocontour lines are drawn at each 1 kcal mol^{-1}. R, TS$^{R\rightarrow I2}$, and I2 designate reactant, transition state of step 1, and intermediate 2, respectively. Energies are in kcal mol^{-1}.

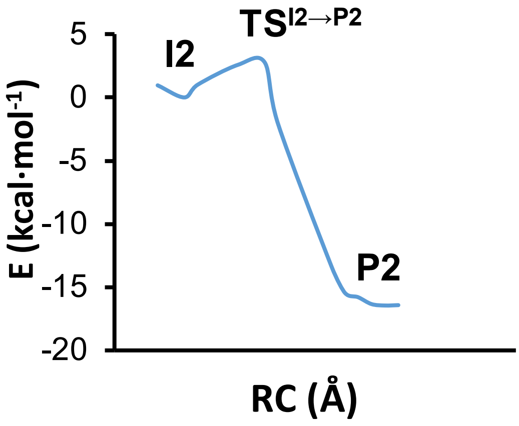

Figure S7. PES at the ωB97X-D/AMBER level of the second step of *Mechanism 2*. I2, $TS^{I2\to P2}$, and P2 designate intermediate 2, transition state of step 2, and product 2, respectively. Energies are in kcal mol^{-1}.

The generated coordinates along the IRCs were used to compute the free energy profiles of both steps. A total of 200 ps of QM/MM MD simulation was done for each window along the IRC path at the temperature of 300 K using the NVT ensemble, maintaining the position of atoms from the QM region fixed during the simulation. For the first step, 31 windows were performed toward the reactant, and 7 windows were performed toward the intermediate, respectively. For the second step, 5 and 26 refer to the intermediate and product, respectively. In Figure S8 is depicted the free energy profile for both steps obtained using free energy perturbation (FEP) methods.[18, 19] The rate-determining step is step 1, and the free energy barrier obtained is 22.8 kcal mol^{-1}. The free energy barrier of the second step is 2.8 kcal mol–1, and the reaction free energy is 1.2 kcal mol^{-1}. This free energy barrier is high compared with the experimental data and other studies,[20-22] and relatively high for an enzymatic reaction; therefore, this reaction mechanism is not viable.

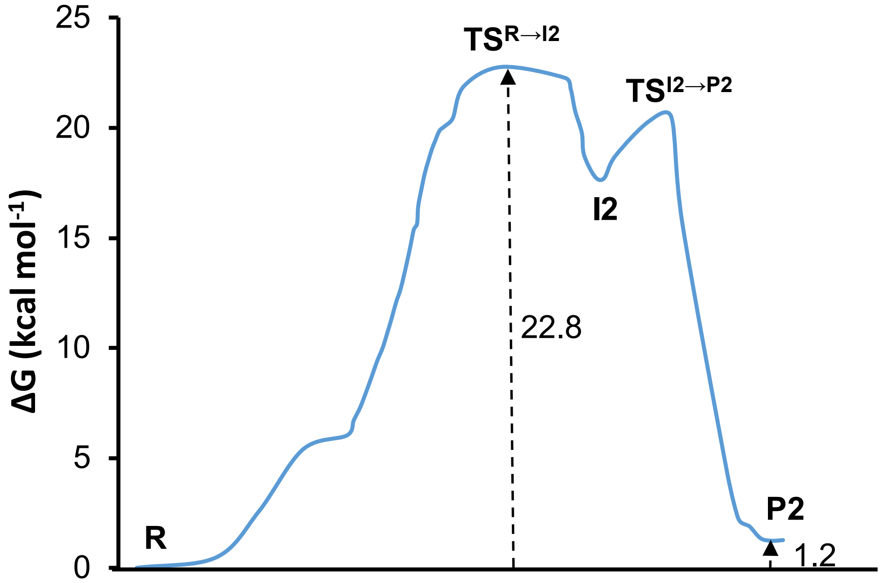

Figure S8. Free energy profile for *Mechanism 2* obtained through free energy perturbation methods at the ωB97X-D/AMBER level. R, $TS^{R\rightarrow I2}$, I2, $TS^{I2\rightarrow P2}$, and P2 designate reactant, transition state of step 1, intermediate 2, transition state of step 2, and product 2, respectively. The free energy barrier for the rate-determining step and the reaction free energy are depicted. Energies are in kcal mol^{-1}.

Mechanism 3. Transfer of the Proton to the α-phosphate Group via a water molecule and the Nucleophilic Attack

Mechanism 3 is depicted in **Figure S9** and **Figure 2** in the main text and consists of the proton transfer from the 3′–OH group of the terminal nucleotide of RNA to the α-phosphate of ATP through a water molecule. Afterwards, the nucleophilic attack takes place.

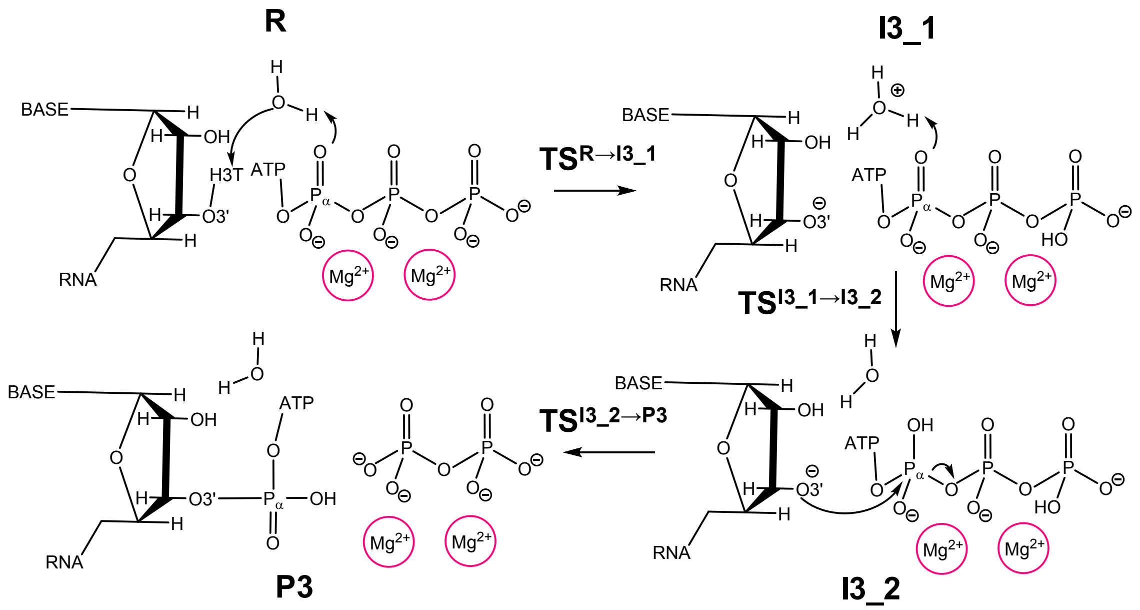

Figure S9. *Mechanism 3* involves proton transfer from the O3′ atom of the terminal nucleotide of the RNA strand to the α-phosphate group via a water molecule, occurring in two steps. A nucleophilic attack on the Pα atom of the incoming nucleotide follows this. R, $TS^{R\rightarrow I3\_1}$, I3_1, $TS^{I3\_1\rightarrow I3\_2}$, I3_2, $TS^{I3\_2\rightarrow P3}$, and P3 denote reactant, transition state of step 1, intermediate 3_1, transition state of step 2, intermediate 3_2, transition state of step 3, and product 3, respectively.

In *Mechanism 3*, the two first steps were studied by means of the exploration of a 2D-PES at the ωB97X-D/AMBER level using as selected distinguished reaction coordinates the antisymmetric combination of the bond breaking and bond forming distances (D(O3′ RNA, H3T RNA) - D(H3T RNA, O WAT) and D(H1 WAT, O WAT) – D (H1 WAT, O2A ATP)) and the number of steps were 40 and 25 for each reaction coordinate, respectively. The force constant used for all the reaction coordinates was 5000 kJ mol^{-1} Å^2, and the step width was 0.1 Å.

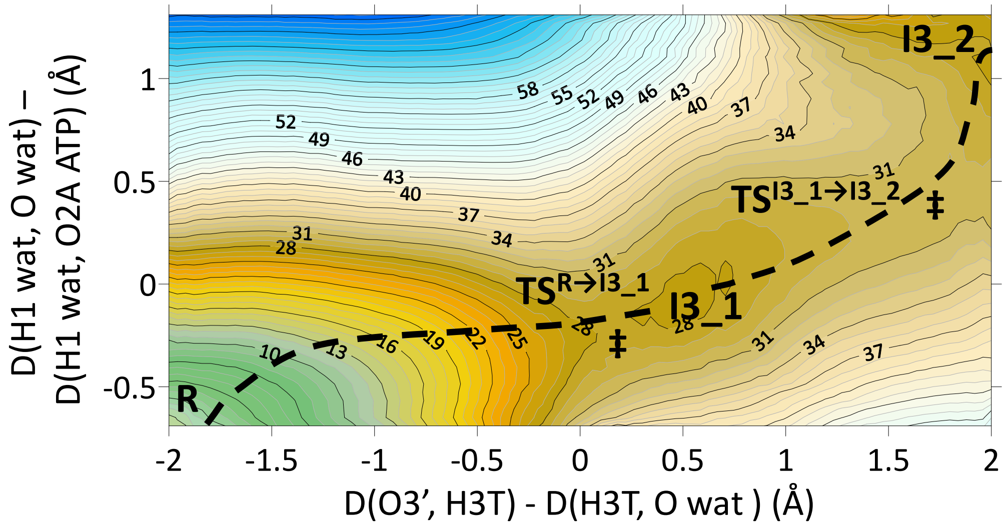

Figure S10. 2D-PES at the ωB97X-D/AMBER level of the two first steps of *Mechanism 3*. Isocontour lines are drawn at intervals of 1 kcal mol^{-1}. R, TS$^{R\rightarrow I3_1}$, I3_1, TS$^{I3_1\rightarrow I3_2}$, and I3_2 designate reactants, transition state of step 1, intermediate 3_1, transition state of step 2, and intermediate 3_2. Energies are in kcal mol^{-1}.

Figure S10 illustrates the 2D potential energy surface (PES) of the first two steps of *Mechanism 3*, computed at the ωB97X-D/AMBER level. Both transition state structures, TS$^{R\rightarrow I3_1}$, and TS$^{I3_1\rightarrow I3_2}$ were localized and characterized at the same computational level. For each transition state, the intrinsic reaction coordinate (IRC)[16, 17] method was applied to trace the minimum energy pathways leading to the reactants and intermediate valleys. The final structures obtained from the IRC were localized, characterized, and confirmed as minima on the PES, corresponding to the reactants (R) and intermediates (I3_1 and I3_2). The potential energy barrier for the first step was 28.6 kcal·mol^{-1}. Consequently, this reaction mechanism was considered energetically infeasible. Therefore, due to this result, we did not proceed with studying this reaction mechanism.

Mechanism 4. Nucleophilic Attack and Proton Transfer to the Pyrophosphate Leaving Group

A bibliographic search on the reaction mechanism catalyzed by RNA polymerases, and more specifically by RdRp from SARS-CoV-2, revealed that not all phosphate groups of the ribonucleoside triphosphate, in this particular study, the ATP was coordinated to Mg^{2+} ions. In particular, the study by Aranda et al.[21] observed that the γ-phosphate was not coordinated to Mg^{2+} ions. In contrast, in the study by Bignon et al.,[22] it was the β-phosphate that was not coordinated to these ions. Upon reviewing our own structures, it was noted that it was possible the β-phosphate was not coordinated to the Mg^{2+} ions. Therefore, a structure was prepared in which the interaction between the β-phosphate and the Mg^{2+} ions was absent, by means of QM/MM MD simulations, using the semiempirical AM1d method[23] to describe the QM region.

From the equilibrated structure, and according to the reaction mechanism proposed by Aranda et al.,[21] *Mechanism 4* was explored. This two-step mechanism is illustrated in **Figure S11** and **Figure 2** in the main text. The first step involves the nucleophilic attack of the deprotonated O3′ atom of the terminal nucleotide of the RNA strand on the Pα atom of the incoming nucleotide (ATP). Thereafter, a proton transfer reaction occurs from the O3′ atom of the newly incorporated terminal nucleotide to the β-phosphate group of the previously formed pyrophosphate leaving group.

The PES of the first step was examined using a single distinguished reaction coordinate that consists of the antisymmetric combination of the bond breaking and bond forming distances (D(O3A ATP, PA ATP) – D(O3′ RNA, PA ATP)), and the number of steps was 40. The PES of the second step was also performed using the antisymmetric combination of the bond breaking and bond forming distances as reaction coordinate (D(O3′ ATP, H3T ATP) – D(H3T ATP, O2B PYR)) where PYR denotes the pyrophosphate leaving group. The number of steps performed was 15. The force constant used for all the reaction coordinates was 5000 kJ $mol^{-1}Å^{2}$, and the step width was 0.1 Å.

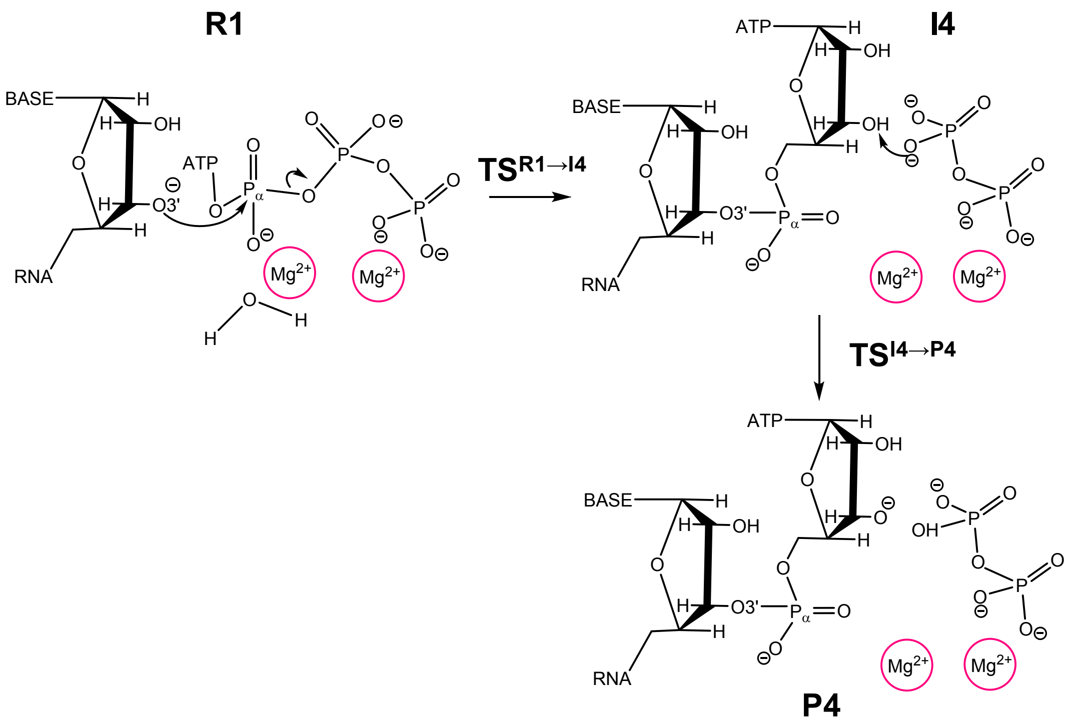

Figure S11. *Mechanism 4* involving the nucleophilic attack of the deprotonated O3′ atom of the terminal RNA nucleotide on the Pα atom of the incoming nucleotide. This is followed by proton transfer from the O3′ atom of the newly incorporated terminal nucleotide to the β-phosphate group of the previously formed pyrophosphate leaving group. R1, $TS^{R1\rightarrow I4}$, I4, $TS^{I4\rightarrow P4}$, and P4 denote reactant, transition state of step 1, intermediate 4, transition state of step 2, and product 4, respectively.

The PESs of both steps of *Mechanism 4*, computed at the ωB97X-D/AMBER level are depicted in **Figure S12**. The $TS^{R1\rightarrow I4}$ structure was localized, and the intrinsic reaction coordinate (IRC)[16, 17] method was applied to trace the minimum energy pathways leading to the reactants and intermediate valleys. However, it was not possible to localize and characterize $TS^{I4\rightarrow P4}$ or the product structure. All the optimizations ended in the intermediate structure. The potential energy barrier of the first step is similar to the results obtained by Orozco and coworkers,[21] and the potential energy profile obtained for step 2 is very similar to the one obtained by these researchers,[21] but the structures of $TS^{I4\rightarrow P4}$ and P4 are unstable, and cannot be localized and characterized. Therefore, we must discard this mechanism.

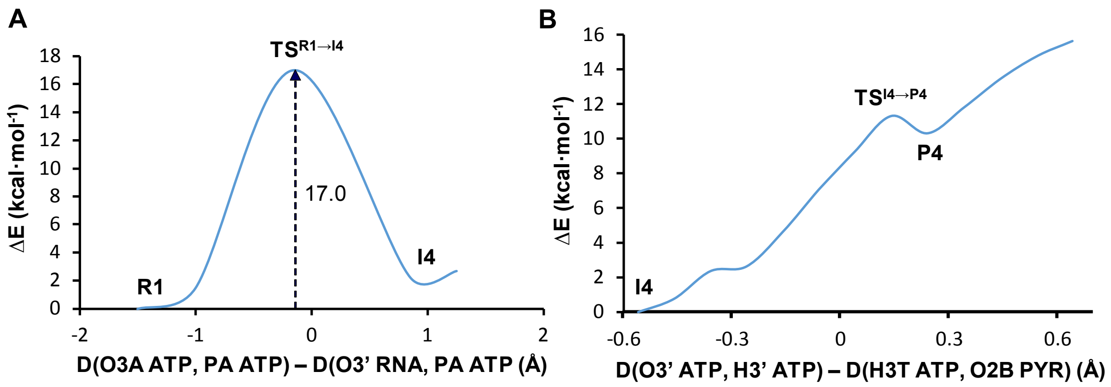

Figure S12. A) PES of step 1, and **B)** PES of step 2 of *Mechanism 4*. Both were performed at the ωB97X-D/MM AMBER level. R1, $TS^{R1\rightarrow I4}$, I4, $TS^{I4\rightarrow P4}$, and P4 denote reactant, transition state of step 1, intermediate 4, transition state of step 2, and product 4, respectively. Energies are in kcal mol^{-1}.

Mechanism 5. Proton Transfer to an OH⁻ group, Nucleophilic Attack and Proton Transfer to the Pyrophosphate Leaving Group

High-resolution X-ray structure of the SARS-CoV-2 RNA-dependent RNA polymerase reveals water molecules coordinated to Mg^{2+} ions that may assist in catalysis or stabilization. Previous computational studies suggest feasible proton transfer from the 3′-OH of the terminal nucleotide to a metal-coordinated or nearby hydroxide ion. Thus, based on the analysis of the R1 structure in Mechanism 4 and in previous experimental and computational studies, we propose Mechanism 5, in which a Mg^{2+}-coordinated hydroxide ion acts as a base to abstract the proton from the 3′-OH of the terminal nucleotide. Consequently, in *Mechanism 5* (**Figure S13** and **Figure 2** in the main text), compared to *Mechanism 4*, a previous step takes place where a proton transfer reaction from the 3′–OH group of the terminal nucleotide to a hydroxide group that is coordinated with a Mg^{2+} ion. Subsequently, the nucleophilic attack of the O3′ atom on the Pα atom occurs, followed by the proton transfer from the water molecule formed in the first step to the γ-phosphate group of the pyrophosphate leaving group. In this mechanism, the hydroxide group coordinated with the Mg^{2+} ion is regenerated to act as a base to abstract the proton of the following terminal nucleotide of the RNA strand, which is just the incorporated nucleotide.

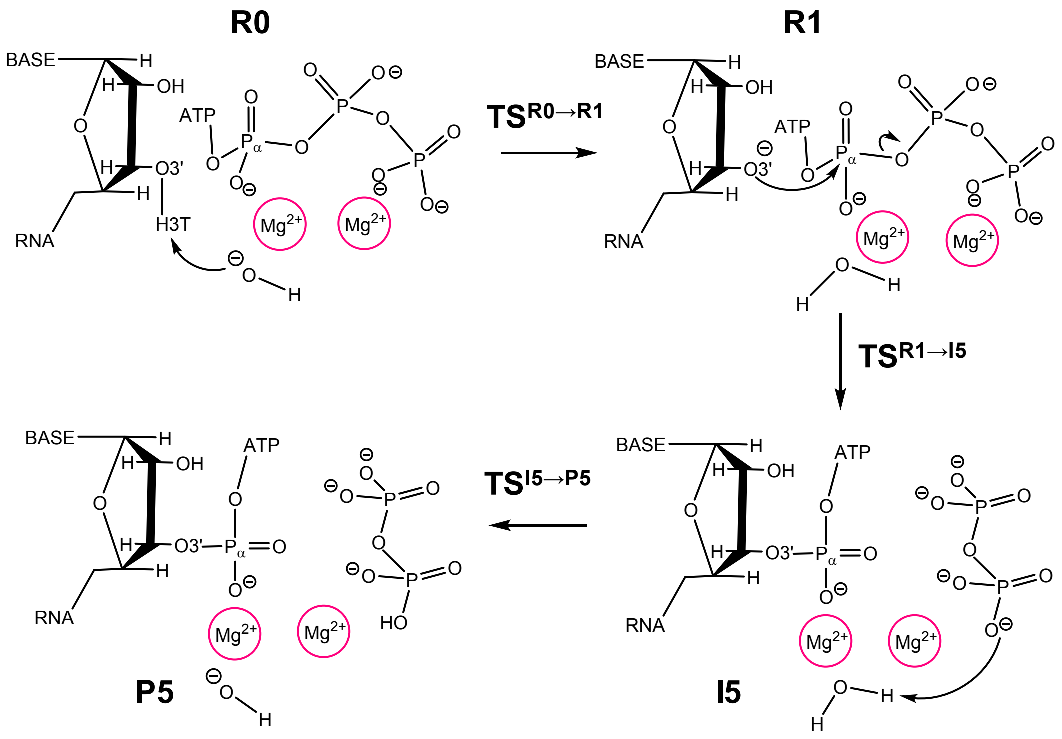

Figure S13. *Mechanism 5* involving the proton transfer from the O3' atom of the terminal RNA nucleotide to the hydroxide group coordinated with an Mg^{2+} atom. This is followed by the nucleophilic attack of the deprotonated O3′ atom on the Pα atom of the incoming nucleotide. Finally, takes place a proton transfer from the water molecule formed in the first step to the γ-phosphate group of the pyrophosphate leaving group. R0, $TS^{R0 \rightarrow R1}$, R1, $TS^{R1 \rightarrow I5}$, I5, $TS^{I5 \rightarrow P5}$ and P5 denote reactant 0, transition state of step 1, reactant 1, transition state of step 2, intermediate 5, transition state of step 3 and product 5, respectively.

The PESs of all the steps of this mechanism were examined at the ωB97X-D/AMBER level using a single distinguished reaction coordinate that consists of the antisymmetric combination of the bond breaking and bond forming distances. In step 1, the reaction coordinate is (D(O3′ RNA, H3T RNA) – D(H3T, O HYD)), where HYD denotes the hydroxide group, and the number of steps was 35. For step 2, the reaction coordinate used is (D(O3A ATP, PA ATP) – D(O3′ RNA, PA ATP)) and the number of steps was 40, while for step 3 is (D(O WAT, H1 WAT) – D(H1 WAT, O2G PPi)), where PPi denotes pyrophosphate group, and the number of steps were 20. The force constant used for all the reaction coordinates was 5000 kJ $mol^{-1}Å^2$, and the step width was 0.1 Å. All the transition state structures were localized and characterized at the same computational level. For each transition state, the intrinsic reaction coordinate (IRC)[16, 17] method was applied to trace the minimum energy pathways leading to the minima valleys. The final structures obtained from the IRC were localized, characterized, and confirmed as minima on the PES, corresponding to the reactants (R0 and R1), intermediate (I5), and products (P5).

The PESs of the three steps of *Mechanism 5* are shown in **Figure S14**. The potential energy barrier for the rate-determining step, which consists of the nucleophilic attack of the O3′ atom of the terminal nucleotide of the RNA strand to the Pα atom of ATP, was found to be 15.7 kcal mol^{-1}. Thus, this mechanism is plausible for an enzymatic reaction.

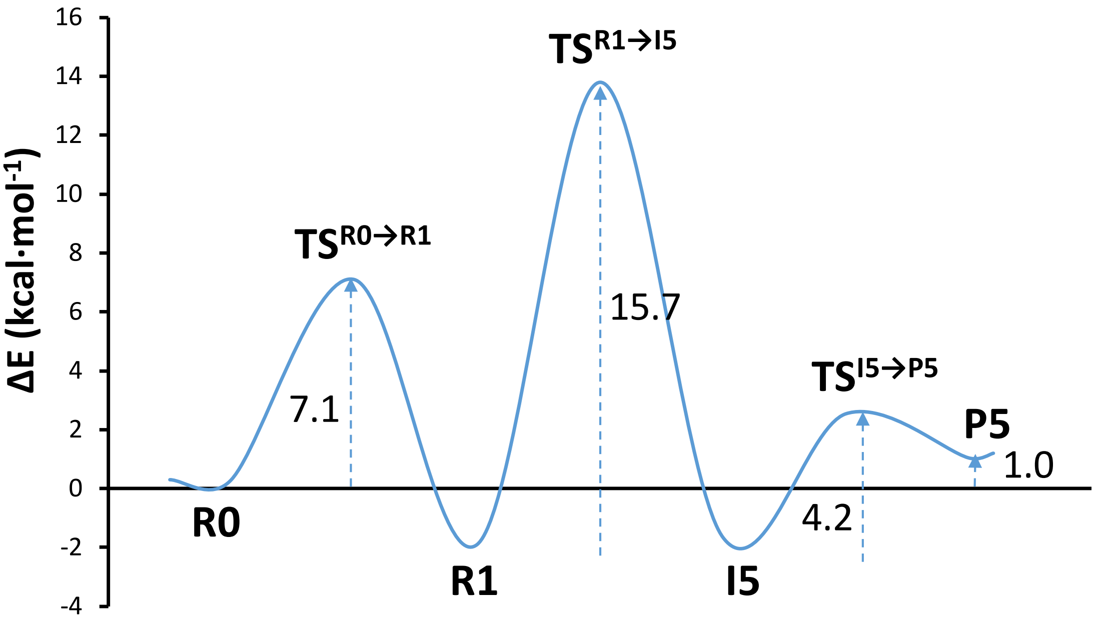

Figure S14. QM/MM cFF potential energy surface (PES) of the three steps of *Mechanism 5* calculated at ωB97X-D/def2-SVP//AMBER. R0, $TS^{R0\rightarrow R1}$, R1, $TS^{R1\rightarrow I5}$, I5, $TS^{I5\rightarrow P5}$, and P5 denote reactant 0, transition state of step 1, reactant 1, transition state of step 2, intermediate 5, transition state of step 3, and product 5, respectively. The potential energy barriers of each step and the reaction energy are shown with arrows and dashed lines. Energies are in kcal mol^{-1}.

The free energy profiles of the three steps were calculated using the coordinates generated along the IRCs. A total of 200 ps of QM/MM MD simulation was performed for each window along the IRC path at a temperature of 300 K using the NVT ensemble, with the positions of atoms in the QM region fixed throughout the simulation. For the first step, 6 and 10 windows were performed toward R0 and R1, respectively. For the second step, 16 and 18 windows were computed toward R1 and I5, respectively, while for the third step, 5 and 2 windows were determined toward I5 and P5, respectively. The free energy landscape (FEL), calculated using the FEP method as described in the Methods section in the main text, is shown in Figure 3, while the optimized DFT/MM TS structures are shown in Figure 4.

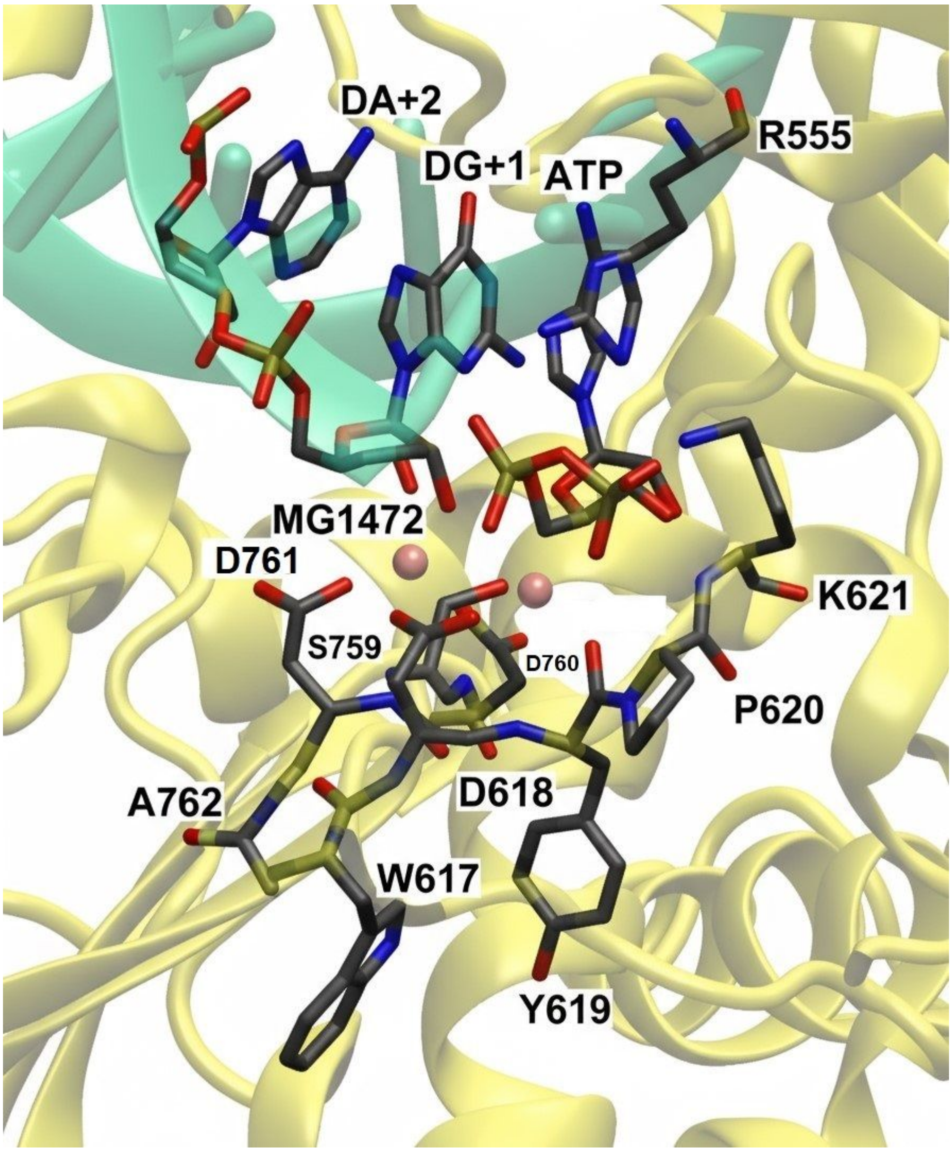

Figure S15. The QM region for the reactant in the extended model comprises 248 QM atoms. The active site's amino acid and nucleotide residues are depicted in a licorice representation, with magnesium cations as pink spheres. Protein and nucleic acid chains are illustrated as yellow and green ribbons, respectively, while hydrogen atoms and water molecules in the active site are omitted for clarity. In addition to the non-standard residues, the QM subsystem includes two magnesium cations (MG1472 and MG1428), residues R555, W617, D618, Y619, P620, K621, C622, S759, D760, D761, A762, nucleotides DG+1 and DA+2, and seven water molecules. Residues and solvent molecules within a 25 Å radius from the active site center (MG1472) are modeled using the AMOEBABIO18 potential. The six pseudobond atoms are the Cα of R555, W617, C622, S759, and A762, and the C5′ of the DA+2 nucleotide, while the Hα, C, O, N, and H atoms of these amino acids and the H5′ atoms plus the phosphate group of the DA+2 nucleotide are considered as the boundary atoms. The smaller QM model (102 QM atoms + 6 pseudobonds) contains the same coordination sphere of the Mg^{2+} ions, preserving the key residues and ligands as in the larger model (see schematic in **Figure S2**).

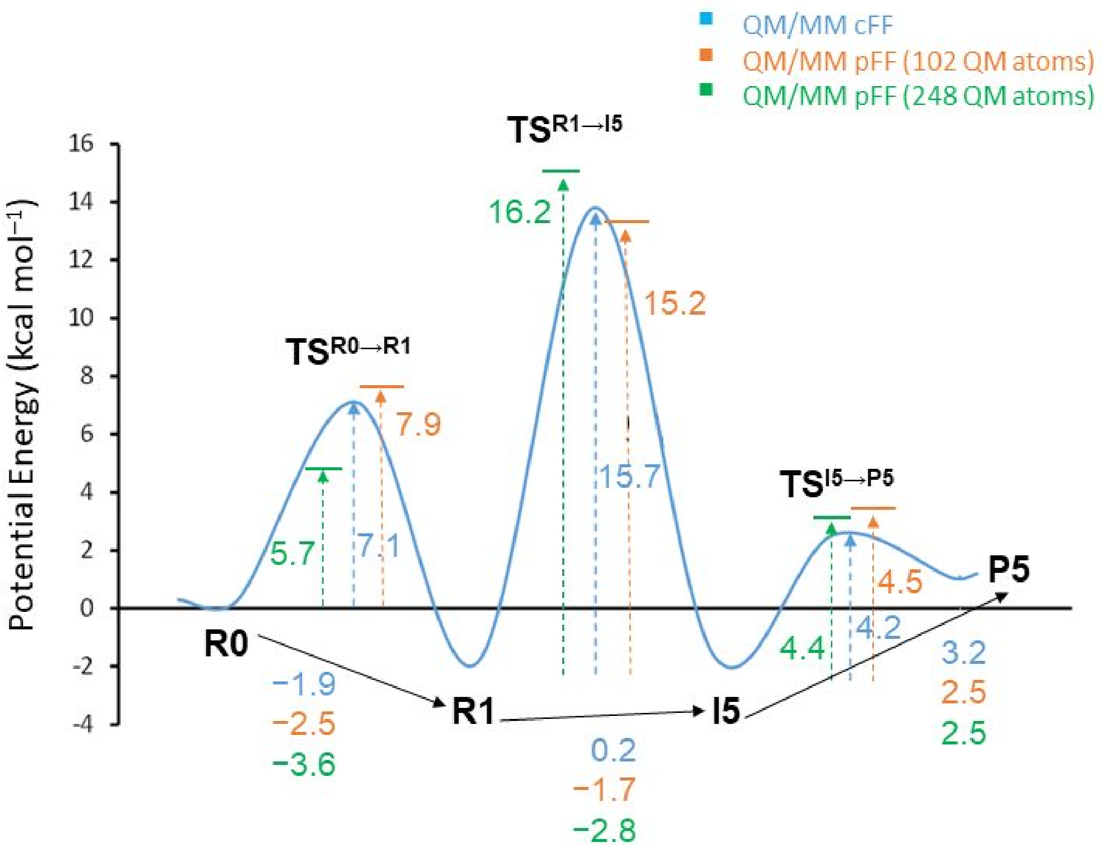

Figure S16. Comparison between the QM/MM PESs calculated with *cFF* (in blue, as previously shown in Figure S14) and the pFF with small (in orange) and large QM region (in green) for *Mechanism 5*. The QM/MM cFF values are computed at *ωB97X-D/def2-SVP//AMBER* level of theory, while the QM/MM pFF energies are calculated at *ωB97X-D/def2-SVP//AMOEBAbio18*. R0, TS$^{R0\rightarrow R1}$, R1, TS$^{R1\rightarrow I5}$, I5, T$^{SI5\rightarrow P5}$, and P5 denote reactant 0, transition state of step 1, reactant 1, transition state of step 2, intermediate 5, transition state of step 3, and product 5, respectively. The potential energy barriers of each step and the reaction energy are shown with arrows and dashed lines. Energies are in kcal mol^{-1}.

Table S1: Results of the ELF populations (e^-) for the critical structures along the polymerization reaction in *Mechanism 5*. Values in plain text are calculated from the polarizable QM/MM optimizations at ωB97X-D/def2-SVP//AMOEBAbio18 level of theory. In contrast, the values in bold italics are calculated from the QM/MM cFF wave functions optimized at ωB97X-D/def2-SVP//AMBER.

Basin	Electron Population (e^-)													
	R0		TS$^{R0\to R1}$		R1		TS$^{R1\to I5}$		I5		TS$^{I5\to P5}$		P5	
V(RNA$^{O3'}$,Mg1,RNA$^{H3'}$)	1.80	***1.80***	-	-	-	-	-	-	-	-	-	-	-	-
V(RNA$^{O3'}$,RNA$^{C3'}$)	1.39	***1.42***	1.42	***1.42***	1.44	***1.46***	1.34	***1.38***	1.33	***1.35***	1.34	***1.36***	1.34	***1.35***
V(RNA$^{O3'}$,Mg1)	2.27<br>2.26	***2.26***<br>***2.26***	2.25<br>2.31<br>1.36	***2.28***<br>***2.31***<br>***1.37***	2.36<br>3.57<br>0.20	***2.41***<br>***3.23***<br>***0.38***	4.75	***2.59***<br>***2.27***	2.50<br>2.44	***2.53***<br>***2.25***	4.74	***2.46***<br>***2.29***	4.75	***4.75***
V(RNA$^{H3'}$,OH$^{-\ O}$)	-	-	-	-	1.78	***1.79***	1.74	***1.76***	1.75	***1.74***	1.73	***1.72***	1.67	***1.69***
V(OH$^{-\ O}$,Mg1,RNA$^{H3'}$)	-	-	0.43	***0.39***	-	-	-	-	-	-	-	-	-	-
V(OH$^{-\ O}$,OH$^{-\ H}$)	1.71	***1.72***	1.71	***1.72***	1.78	***1.77***	1.78	***1.76***	1.77	***1.77***	-	-	-	-
V(OH$^{-\ O}$)	*2.73*	***2.75***<br>***1.45***	2.92	***2.89***	2.39	***2.39***	2.29	***2.36***	2.31	***2.34***	2.55<br>1.27	***3.83***	4.03	***3.81***
V(OH$^{-\ O}$,Mg1)	*1.88*<br>*1.44*	***1.83***	1.80<br>1.29	***1.84***<br>***1.26***	1.94	***1.98***	2.09	***2.04***	2.07	***2.08***	2.14	***2.15***	2.07	***2.24***
V(RNA$^{O3'}$–ATP$^{P\alpha}$)	-	-	-	-	-	-	-	-	1.56	***1.54***	1.56	***1.55***	1.55	***1.54***
V(ATP$^{P\alpha}$,ATP$^{O3\alpha}$)	1.53	***1.52***	1.54	***1.50***	1.51	***1.49***	-	-	-	-	-	-	-	-
V(ATP$^{O3\alpha}$,ATP$^{P\beta}$)	1.51	***1.48***	1.51	***1.49***	1.51	***1.48***	1.80	***1.70***	1.78	***1.77***	1.79	***1.79***	1.82	***1.80***
V(ATP^{O3A})	4.75	***4.80***	4.74	***4.80***	4.75	***4.82***	3.97	***3.89***	4.06<br>1.95	***6.01***	3.83<br>2.17	***3.48***<br>***2.44***	3.69<br>2.28	***6.00***
V(RNA$^{O3'}$,ATP$^{P\alpha}$,ATP$^{O3\alpha}$)	-	-	-	-	-	-	2.03	***2.08***	-	-	-	-	-	-
V(Mg2,ATP$^{P\gamma}$,ATP$^{O3\gamma}$)	-	-	1.68	***1.65***	1.65	***1.61***	1.56	***1.53***	1.56	***1.49***	-	-	1.88	***1.85***
V(ATP$^{O3\gamma}$,Mg2)	2.25	***2.36***	2.13	***2.36***	2.19	***2.32***	1.91	***2.11***	2.16	***2.21***	1.97	***2.01***	2.08	***1.97***
V(ATP$^{O3\gamma}$)	3.88	***3.80***	4.00	***3.80***	3.96	***3.86***	4.29	***4.13***	4.04	***4.04***	4.15	***4.11***	2.53	***2.68***
V(ATP$^{P\gamma}$,ATP$^{O3\gamma}$,OH$^{-\ H}$)	-	-	-	-	-	-	-	-	-	-	1.50	***1.46***	-	-
V(ATP$^{O3\gamma}$,OH$^{-\ H}$)	-	-	-	-	-	-	-	-	-	-	-	-	1.40	***1.41***

- RNA$^{O3'}$ and RNA$^{H3'}$ refer to the oxygen and hydrogen atoms of the terminal nucleotide's 3'–OH group (see **Figure 6** in the main text).
- Mg1 is the catalytic Mg^{2+} ion coordinated to the terminal nucleotide, while Mg2 is the auxiliary Mg^{2+} ion surrounded by the ATP's triphosphate group.
- OH$^{-\ O}$ and OH$^{-\ H}$ refer to the oxygen and hydrogen atoms of the hydroxide group coordinated with the Mg1ion.
- ATP$^{P\alpha}$, ATP$^{O3\alpha}$, ATP$^{P\gamma}$, and ATP$^{O3\gamma}$ refer to the pertinent atoms of the ATP incoming nucleotide (see **Figure 6** in the main text).

Table S2: Results of the dipole moment magnitude (|μ| in a.u.) for the critical structures along the polymerization reaction in *Mechanism 5*. Values in plain text are calculated from the polarizable QM/MM optimizations at ωB97X-D/def2-SVP//AMOEBAbio18 level of theory. In contrast, the values in bold italics are calculated from the QM/MM cFF wave functions optimized at ωB97X-D/def2-SVP//AMBER.

Basin	Dipole Moment Magnitude (|μ|)													
	R0		$TS^{R0\rightarrow R1}$		R1		$TS^{R1\rightarrow I5}$		I5		$TS^{I5\rightarrow P5}$		P5	
$V(RNA^{O3'},Mg1,RNA^{H3'})$	1.33	***1.15***	-	-	-	-	-	-	-	-	-	-	-	-
$V(RNA^{O3'},RNA^{C3'})$	0.17	***0.14***	0.18	***0.16***	0.12	***0.11***	0.17	***0.13***	0.14	***0.14***	0.21	***0.20***	0.15	***0.12***
$V(RNA^{O3'},Mg1)$	0.99<br>0.77	***0.87***<br>***0.61***	0.97<br>0.79<br>0.54	***0.87***<br>***0.78***<br>***0.39***	1.97<br>0.80<br>0.10	***1.71***<br>***0.73***<br>***0.04***	2.97	***1.13***<br>***1.07***	2.07<br>1.25	***1.77***<br>***1.32***	3.41	***1.93***<br>***1.23***	3.52	***3.40***
$V(RNA^{H3'},OH^{-\ O})$	-	-	-	-	1.12	***1.09***	1.06	***0.92***	1.03	***0.96***	0.96	***0.94***	0.76	***0.75***
$V(OH^{-\ O},Mg1,RNA^{H3'})$	-	-	0.05	***0.03***	-	-	-	-	-	-	-	-	-	-
$V(OH^{-\ O},OH^{-\ H})$	0.86	***0.79***	0.90	***0.90***	1.08	***1.07***	1.14	***1.14***	1.19	***1.12***	-	-	-	-
$V(OH^{-\ O})$	*0.99*	***0.89***<br>***0.40***	1.32	***1.17***	0.96	***1.03***	0.96	***0.84***	0.94	***0.75***	0.96<br>0.26	***0.95***	1.39<br>0.41	***0.98***<br>***0.29***
$V(OH^{-\ O},Mg1)$	*0.42*<br>*0.37*	***0.39***	0.59<br>0.37	***0.53***<br>***0.31***	0.82	***0.74***	0.80	***0.64***	0.84	***0.74***	0.64	***0.59***	0.58	***0.57***
$V(RNA^{O3'}–ATP^{P\alpha})$	-	-	-	-	-	-	-	-	0.15	***0.06***	0.10	***0.14***	0.12	***0.08***
$V(ATP^{P\alpha},ATP^{O3\alpha})$	0.07	***0.04***	0.13	***0.04***	0.14	***0.11***	-	-	-	-	-	-	-	-
$V(ATP^{O3\alpha},ATP^{P\beta})$	0.15	***0.07***	0.06	***0.08***	0.11	***0.11***	0.22	***0.17***	0.32	***0.20***	0.33	***0.33***	0.48	***0.26***
$V(ATP^{O3A})$	4.63	***4.45***	4.29	***2.36***	4.37	***2.89***	4.35	***3.43***	3.43<br>0.92	***4.09***	3.33<br>0.78	***2.84***<br>***0.84***	3.19<br>0.74	***3.63***
$V(RNA^{O3'},ATP^{P\alpha},ATP^{O3\alpha})$	-	-	-	-	-	-	0.11	***0.09***	-	-	-	-	-	-
$V(Mg2,ATP^{P\gamma},ATP^{O3\gamma})$	-	-	0.26	***0.14***	0.17	***0.15***	0.19	***0.14***	0.15	***0.12***	-	-	0.04	***0.05***
$V(ATP^{O3\gamma},Mg2)$	0.77	***0.70***	0.80	***0.71***	0.78	***0.69***	0.84	***0.55***	0.71	***0.65***	0.96	***0.91***	0.86	***0.85***
$V(ATP^{O3\gamma})$	2.55	***2.38***	2.75	***2.36***	2.49	***2.40***	3.27	***3.06***	2.97	***2.86***	1.53	***1.47***	1.25	***0.93***
$V(ATP^{P\gamma},ATP^{O3\gamma},OH^{-\ H})$	-	-	-	-	-	-	-	-	-	-	0.39	***0.27***	-	-
$V(ATP^{O3\gamma},OH^{-\ H})$	-	-	-	-	-	-	-	-	-	-	-	-	1.18	***1.23***

REFERENCES

1. Frisch, M. J.; Trucks, G. W.; Schlegel, H. B.; Scuseria, G. E.; Robb, M. A.; Cheeseman, J. R.; Scalmani, G.; Barone, V.; Petersson, G. A.; Nakatsuji, H.; Li, X.; Caricato, M.; Marenich, A. V.; Bloino, J.; Janesko, B. G.; Gomperts, R.; Mennucci, B.; Hratchian, H. P.; Ortiz, J. V.; Izmaylov, A. F.; Sonnenberg, J. L.; Williams; Ding, F.; Lipparini, F.; Egidi, F.; Goings, J.; Peng, B.; Petrone, A.; Henderson, T.; Ranasinghe, D.; Zakrzewski, V. G.; Gao, J.; Rega, N.; Zheng, G.; Liang, W.; Hada, M.; Ehara, M.; Toyota, K.; Fukuda, R.; Hasegawa, J.; Ishida, M.; Nakajima, T.; Honda, Y.; Kitao, O.; Nakai, H.; Vreven, T.; Throssell, K.; Montgomery Jr., J. A.; Peralta, J. E.; Ogliaro, F.; Bearpark, M. J.; Heyd, J. J.; Brothers, E. N.; Kudin, K. N.; Staroverov, V. N.; Keith, T. A.; Kobayashi, R.; Normand, J.; Raghavachari, K.; Rendell, A. P.; Burant, J. C.; Iyengar, S. S.; Tomasi, J.; Cossi, M.; Millam, J. M.; Klene, M.; Adamo, C.; Cammi, R.; Ochterski, J. W.; Martin, R. L.; Morokuma, K.; Farkas, O.; Foresman, J. B.; Fox, D. J. *Gaussian 09* Wallingford, CT, 2009.
2. Martí, S.; Moliner, V.; Tuñón, I., Improving the QM/MM Description of Chemical Processes: A Dual Level Strategy To Explore the Potential Energy Surface in Very Large Systems. *Journal of Chemical Theory and Computation* **2005**, 1, 1008-1016.
3. Chai, J.-D.; Head-Gordon, M., Long-range corrected hybrid density functionals with damped atom–atom dispersion corrections. *Physical Chemistry Chemical Physics* **2008**, 10, 6615-6620.
4. Maier, J. A.; Martinez, C.; Kasavajhala, K.; Wickstrom, L.; Hauser, K. E.; Simmerling, C., ff14SB: Improving the Accuracy of Protein Side Chain and Backbone Parameters from ff99SB. *Journal of Chemical Theory and Computation* **2015**, 11, 3696-3713.
5. Wang, J.; Cieplak, P.; Kollman, P. A., How well does a restrained electrostatic potential (RESP) model perform in calculating conformational energies of organic and biological molecules? *Journal of computational chemistry* **2000**, 21, 1049-1074.
6. Pérez, A.; Marchán, I.; Svozil, D.; Sponer, J.; Cheatham, T. E.; Laughton, C. A.; Orozco, M., Refinement of the AMBER force field for nucleic acids: improving the description of α/γ conformers. *Biophysical journal* **2007**, 92, 3817-3829.
7. Zgarbová, M.; Otyepka, M.; Sponer, J.; Mladek, A.; Banas, P.; Cheatham III, T. E.; Jurecka, P., Refinement of the Cornell et al. nucleic acids force field based on reference quantum chemical calculations of glycosidic torsion profiles. *Journal of chemical theory and computation* **2011**, 7, 2886-2902.
8. Steinbrecher, T.; Latzer, J.; Case, D., Revised AMBER parameters for bioorganic phosphates. *Journal of chemical theory and computation* **2012**, 8, 4405-4412.
9. Peters, M. B.; Yang, Y.; Wang, B.; Fusti-Molnar, L.; Weaver, M. N.; Merz Jr, K. M., Structural survey of zinc-containing proteins and development of the zinc AMBER force field (ZAFF). *Journal of chemical theory and computation* **2010**, 6, 2935-2947.
10. Jorgensen, W. L.; Chandrasekhar, J.; Madura, J. D.; Impey, R. W.; Klein, M. L., Comparison of Simple Potential Functions for Simulating Liquid Water. *J. Chem. Phys.* **1983**, 79, 926.
11. Allnér, O.; Nilsson, L.; Villa, A., Magnesium ion–water coordination and exchange in biomolecular simulations. *Journal of chemical theory and computation* **2012**, 8, 1493-1502.
12. Florián, J.; Goodman, M. F.; Warshel, A., Computer simulation of the chemical catalysis of DNA polymerases: discriminating between alternative nucleotide insertion mechanisms for T7 DNA polymerase. *J Am Chem Soc* **2003**, 125, 8163-77.
13. Cisneros, G. A.; Perera, L.; García-Díaz, M.; Bebenek, K.; Kunkel, T. A.; Pedersen, L. G., Catalytic mechanism of human DNA polymerase lambda with Mg2+ and Mn2+ from ab initio quantum mechanical/molecular mechanical studies. *DNA Repair (Amst)* **2008**, 7, 1824-34.
14. Rungrotmongkol, T.; Mulholland, A. J.; Hannongbua, S., QM/MM simulations indicate that Asp185 is the likely catalytic base in the enzymatic reaction of HIV-1 reverse transcriptase. *MedChemComm* **2014**, 5, 593-596.

15. Wilson, K. A.; Fernandes, P. A.; Ramos, M. J.; Wetmore, S. D., Exploring the Identity of the General Base for a DNA Polymerase Catalyzed Reaction Using QM/MM: The Case Study of Human Translesion Synthesis Polymerase η. *ACS Catalysis* **2019**, 9, 2543-2551.
16. Fukui, K.-i., Formulation of the reaction coordinate. *The Journal of Physical Chemistry* **1970**, 74, 4161-4163.
17. Fukui, K., The path of chemical reactions - the IRC approach. *Accounts of Chemical Research* **1981**, 14, 363-368.
18. Bash, P. A.; Field, M. J.; Karplus, M., Free energy perturbation method for chemical reactions in the condensed phase: a dynamic approach based on a combined quantum and molecular mechanics potential. *Journal of the American Chemical Society* **1987**, 109, 8092-8094.
19. Świderek, K.; Tuñón, I.; Martí, S.; Moliner, V.; Bertrán, J., Role of Solvent on Nonenzymatic Peptide Bond Formation Mechanisms and Kinetic Isotope Effects. *Journal of the American Chemical Society* **2013**, 135, 8708-8719.
20. Kellinger, M. W.; Ulrich, S.; Chong, J.; Kool, E. T.; Wang, D., Dissecting Chemical Interactions Governing RNA Polymerase II Transcriptional Fidelity. *Journal of the American Chemical Society* **2012**, 134, 8231-8240.
21. Aranda, J.; Wieczór, M.; Terrazas, M.; Brun-Heath, I.; Orozco, M., Mechanism of reaction of RNA-dependent RNA polymerase from SARS-CoV-2. *Chem Catalysis* **2022**, 2, 1084-1099.
22. Bignon, E.; Monari, A., Modeling the Enzymatic Mechanism of the SARS-CoV-2 RNA-Dependent RNA Polymerase by DFT/MM-MD: An Unusual Active Site Leading to High Replication Rates. *Journal of Chemical Information and Modeling* **2022**, 62, 4261-4269.
23. Nam, K.; Cui, Q.; Gao, J.; York, D. M., Specific reaction parametrization of the AM1/d Hamiltonian for phosphoryl transfer reactions: H, O, and P atoms. *J. Chem. Theory Comput.* **2007**, 3, 486−504.
